# Advanced photocatalytic materials based degradation of micropollutants and their use in hydrogen production – a review

**DOI:** 10.1039/d4ra01307g

**Published:** 2024-05-02

**Authors:** Surendar Balu, Dhanraj Ganapathy, Sandeep Arya, Raji Atchudan, Ashok K. Sundramoorthy

**Affiliations:** a Department of Prosthodontics, Centre for Nano-Biosensors, Saveetha Dental College and Hospitals, Saveetha Institute of Medical & Technical Sciences, Saveetha University Chennai 600077 Tamil Nadu India ashok.sundramoorthy@gmail.com; b Department of Physics, University of Jammu 180006 Jammu Jammu and Kashmir India; c School of Chemical Engineering, Yeungnam University 38541 Gyeongsan Republic of Korea

## Abstract

The use of pharmaceuticals, dyes, and pesticides in modern healthcare and agriculture, along with expanding industrialization, heavily contaminates aquatic environments. This leads to severe carcinogenic implications and critical health issues in living organisms. The photocatalytic methods provide an eco-friendly solution to mitigate the energy crisis and environmental pollution. Sunlight-driven photocatalytic wastewater treatment contributes to hydrogen production and valuable product generation. The removal of contaminants from wastewater through photocatalysis is a highly efficient method for enhancing the ecosystem and plays a crucial role in the dual-functional photocatalysis process. In this review, a wide range of catalysts are discussed, including heterojunction photocatalysts and various hybrid semiconductor photocatalysts like metal oxides, semiconductor adsorbents, and dual semiconductor photocatalysts, which are crucial in this dual function of degradation and green fuel production. The effects of micropollutants in the ecosystem, degradation efficacy of multi-component photocatalysts such as single-component, two-component, three-component, and four-component photocatalysts were discussed. Dual-functional photocatalysis stands out as an energy-efficient and cost-effective method. We have explored the challenges and difficulties associated with dual-functional photocatalysts. Multicomponent photocatalysts demonstrate superior efficiency in degrading pollutants and producing hydrogen compared to their single-component counterparts. Dual-functional photocatalysts, incorporating TiO_2_, g-C_3_N_4_, CeO_2_, metal organic frameworks (MOFs), layered double hydroxides (LDHs), and carbon quantum dots (CQDs)-based composites, exhibit remarkable performance. The future of synergistic photocatalysis envisions large-scale production facilitate integrating advanced 2D and 3D semiconductor photocatalysts, presenting a promising avenue for sustainable and efficient pollutant degradation and hydrogen production from environmental remediation technologies.

## Abstract 2

The future directions of photocatalytic degradation of micropollutants (pharmaceuticals, dyes, pesticides, *etc.*) and its role in green fuel production from wastewater are discussed with several examples of modern photocatalysts.

## Graphical abstract

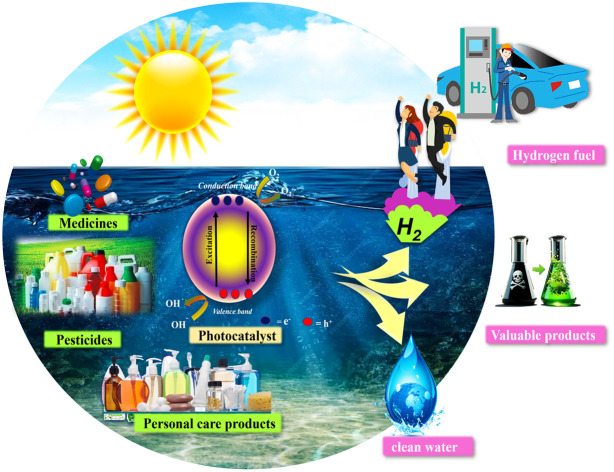

## 1. Introduction

The increasing prevalence of micropollutants in the environment has generated mounting apprehension over their adverse effects on ecosystems and human well-being.^1^ The growth of micropollutants has increased significantly in the wake of the COVID-19 pandemic, leading to significant implications for various aspects of our ecology. Micropollutants also had substantial impacts on human health. The existence of micropollutants, a diverse group of chemical compounds originating from pharmaceutical wastewater, pesticides, personal care items, and various other contaminants, presents a major challenge owing to their hazardous nature and resistance to traditional wastewater disposal techniques. Several medicinal products are incapable of being biodegradable because of their very stable chemical structures. Several antibiotics and analgesics preserve 90% of their active chemical components in an aqueous media, and many therapeutic drugs remain quite active long beyond their expiration date (*i.e.*, 28–40 years).^2,3^ Secondary pollution is on the ascent due to the proliferation of antibiotic residues in the environment. This phenomenon can trigger several unintended repercussions, resulting in the formation of enduring byproducts that prove challenging to decompose, even with contemporary wastewater treatment methods.^4^ The widespread toxicity and threat that antimicrobial resistance (AMR) poses to living ecosystems have made its detection, elimination, and degradation in water systems a pressing concern all over the globe. With the ongoing rise of the global population, there has been a corresponding increase in the discharge of harmful pollutants into aquatic environments and terrestrial ecosystems. To address this challenge, it is essential to use new and sustainable technologies that can effectively eliminate micropollutants from water sources. In the world of water treatment, conventional methods have long been relied upon to address the presence of micropollutants.^5^ The widespread elimination of these pollutants from water sources is rapidly demonstrated through various treatment methods such as coagulation, sedimentation, and activated carbon adsorption.^6^ Micropollutants, which encompass a wide range of substances such as pharmaceuticals, personal care products, and pesticides, pose a significant challenge to water treatment facilities. These compounds are typically present in low concentrations, making their removal as a difficult process. Coagulation, a commonly employed process, involves the addition of chemicals to water to facilitate the aggregation of particles and contaminants.^7^ While effective in removing larger particles and some dissolved substances, coagulation may not be sufficient to adequately address micropollutants. Some of these compounds often exhibit resistance to coagulation agents, leading to incomplete removal. However, they have certain drawbacks such as limited efficiency, a large expenditure of energy, the possibility of producing extra harmful by-products, and also the previously mentioned technologies were not completely removing micropollutants from wastewater. The utilization of advanced oxidation processes (AOPs) is a viable solution to address this significant issue. AOPs provide many benefits, mostly attributed to the production of highly reactive hydroxyl radicals. These radicals possess enormous oxidation potential and may effectively decompose the active micropollutants contained in aquatic environments.^8^ In recent years, employing the technique of photocatalytic degradation has come forward as a possible and ecologically sustainable method for addressing the issues caused by micropollutants. Photocatalysis is a phenomenon that exploits the energy of light and certain photocatalysts to begin chemical transformations.^9^ Upon exposure to light, these photocatalysts produce extremely reactive radicals, such as hydroxyl radicals (˙OH) and superoxide radicals (˙O_2_^−^), which have powerful oxidizing characteristics and can efficiently degrade organic contaminants into innocuous compounds like carbon dioxide (CO_2_) and water (H_2_O). The radicals exhibit a high degree of efficacy in the process of eliminating the chemical bonds of micropollutants, resulting in their subsequent degradation into benign chemicals or full mineralization into less complex molecules.^10^ The effectiveness of photocatalytic degradation is influenced by several parameters, such as the selection of the photocatalyst, the characteristics of the light source, the pH of the solution, and the presence of coexisting compounds. Researchers have conducted thorough investigations into tuning these parameters to enhance the performance of photocatalytic systems and reach the effective degradation of micropollutants.^11^

The field of photocatalytic technique can facilitate the decomposition of contaminants in water *via* the process of photocatalytic decomposition. Additionally, it has the further advantage of producing hydrogen, while also mitigating the cost escalation and environmental contamination associated with the usage of a sacrificial agent. In contrast to traditional studies that treat hydrogen production and pollutant degradation as separate processes, dual-function photocatalysis necessitates a photocatalyst capable of simultaneously accommodating both reactions.^12^ This presents a significant challenge and is the central focus of current research on synergistic contaminants oxidation in photocatalytic hydrogen generation. The complexity arises from the fact that various pollutants exhibit distinct reaction mechanisms and require different environmental conditions. Furthermore, catalyst materials possess diverse chemical and physical characteristics, further complicating the task.^13^ The integration of photocatalytic hydrogen generation and pollutant degradation not only facilitates hydrogen production but also allows for the tailoring of reaction parameters and catalytic materials to specific pollutant categories or product specifications.^14^ This dual functionality enables the comprehensive elimination of contaminants or the conversion of valuable goods. The concept of bifunctional synergy underscores the mutual selectivity between the photocatalyst and the pollutant. To optimize the process and achieve superior outcomes in hydrogen generation and pollutant degradation, it is crucial to carefully align the catalyst, pollutant, and ambient parameters. This requires a harmonious match between the catalyst, pollutant, and the surrounding medium. The effectiveness of this approach relies on finding the right balance among the catalyst, pollutant, and environmental factors.^15^ This overview of the literature endeavors to provide a complete function of photocatalysis in the degradation of micropollutants and its potential in the development of green fuel. This paper provides a thorough examination of current research on photocatalytic hydrogen generation with synergistic pollutant degradation. Recent advancements in catalyst materials, composites, microstructures, pollutant characteristics, co-existing chemicals, and treatment requirements are the primary focus. The assessment considers both catalyst and pollutant features, with a specific emphasis on identifying significant challenges and promising developments in the respective fields of study. Furthermore, an examination will be conducted on the obstacles and potential opportunities linked to the use of photocatalysis as an environmentally friendly technique for mitigating environmental pollution and promoting the production of renewable fuels and valuable byproducts.

## 2. The effects of micropollutants in the ecosystem

**Fig. 1:**
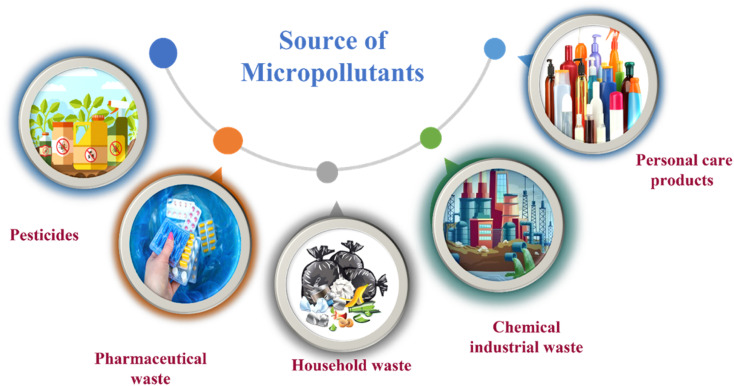
Representation of the various origins of micropollutants in the ecosystem.

Micropollutants have become a global issue in both terrestrial and water-based environments. Industrial wastewater, agricultural runoff, medical industry effluents, household waste, and personal care products are the significant sources of micropollutants in the environment.^16^ Wastewater from large-scale pharmaceutical, pesticide, and other chemical companies contributed significantly to micropollutant contamination. Runoff from farming and livestock-rearing regions are also a major source of micropollutants, notably pesticides used to boost production, as well as hormonal steroids and antibiotics used for animal upkeep.^17^ Moreover, a multitude of micropollutants and their subsequent transformation products have the potential to enter agricultural areas *via* the use of treated wastewater for irrigation purposes. Consequently, the bodies of water that receive these wastewaters become polluted by these chemicals.^18^ Other sources of micropollutants include wastewater treatment plants, discharges originating from landfills, industrial waste streams, and septic tanks. The presence of micropollutants, such as pesticides, pharmaceuticals, steroid hormones, and personal care products (Fig. 1), are significantly contributed by domestic wastewater.^19^ Small quantities of those substances are also provided by their domestic applications as well as usage in several kinds of valuable products. For example, pharmaceutical medications that are consumed orally are eventually eliminated by the human body after digestion in feces and urine, which, if left untreated, reveal their existence in the environment.^20^

Considering the fact that all micropollutants can be categorized under the same category based on their characteristics, they all pose a threat to both human wellness and the ecosystem.^21^ Studies have shown various kinds of effects associated with micropollutants, including both immediate and prolonged toxicological impacts as well as disruptions to the endocrine system resulting from exposure to these substances. Several of these chemical substances have a tendency to disrupt the normal functioning of the endocrine system, leading to the development of cancerous tumors and other congenital abnormalities in infants, at certain dosage levels.^22^ There have been many health concerns associated with possible exposure to micropollutants. These include an increased risk of testicular and thyroid cancers in males, developmental abnormalities in children and babies, breast cancer, diabetes/metabolic syndrome, and fertility failures.^23^ Frequent exposure to these compounds also leads to the development of antibiotic resistance in some bacteria, hence complicating their treatment. Extended periods of exposure may also result in the phenomenon of bioaccumulation within the environment.^24^ Thus, pollution of the environment with micropollutants is discovered to have a detrimental impact on the health of all living species. Given the detrimental health impacts of these micropollutants, sufficient technologies, such as advanced oxidation processes such as photocatalytic degradation, have to be implemented to prevent their release into the environmental system.

## 3. Photocatalysis: principles and mechanisms

**Fig. 2:**
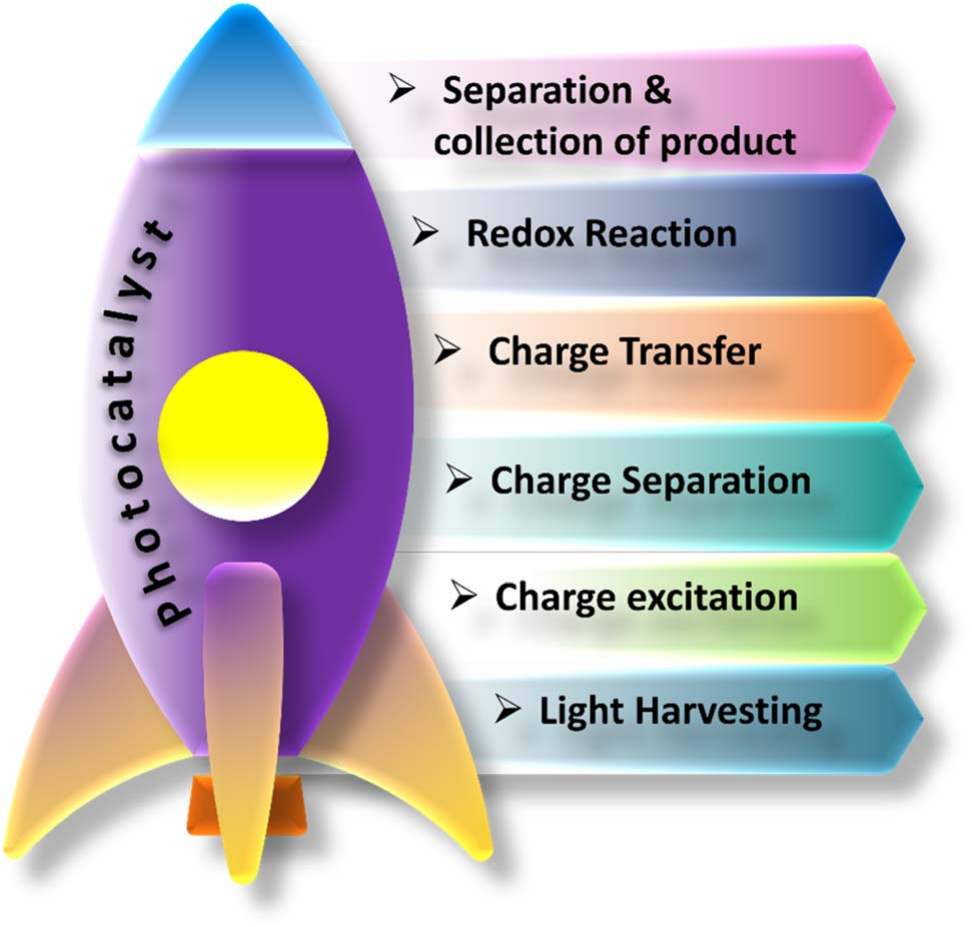
Representation of the various stages associated with the applications of photocatalysts.

Photocatalysis is a branch of research that utilizes light energy to drive chemical reactions. It makes use of photocatalysts, which are compounds that absorb light and induce certain chemical processes. Photocatalysis has gotten a lot of interest because of its potential uses in a wide range of fields, which include remediation of environmental damage and energy conversion.^25^ A photocatalyst is a substance that can absorb light energy, generally in the form of ultraviolet (200–400 nm), visible light (400–800 nm), and infrared. Fig. 2 shows the list of various steps involved in photocatalytic reactions. Electrons in the photocatalyst are excited, forming reactive species such as pairs of electrons and holes or free radicals.^26^ Photocatalysis represents a photoinduced advanced oxidation process that takes place on a semiconductor surface. The tenet of photocatalysis was initially proposed by Fujishima and Honda during the 1970's and subsequently called the Honda–Fujishima effect.^27^ The process starts possibly when a wavelength of light energy (*hv*) that equal or exceed the bandgap energy of a semiconductor is present. Upon illumination, the valence band (VB) electrons undergo excitation and transition to the conduction band (CB), while the positively charged holes (h^+^) remain in the valence band (VB). Both electrons (e^−^) and holes (h^+^) are capable of migrating to the surfaces of the photocatalyst.^28^ The remaining holes participate in the oxidation of water, resulting in the generation of hydroxyl radicals (˙OH). Electrons located in the conduction band (CB) undergo a reaction with atmospheric oxygen, producing superoxide radicals. Photocatalysis is one of the significant ways to degrade organic pollutants and hydrogen production. More potential and efficient pollutant degrading ability is accomplished by using spontaneous and non-spontaneous processes to enhance the entire process. As a result of Fujishima and Honda's discovery, electrochemical photolysis of water at semiconductor electrodes, was reported.^29^

1 TiO_2_ + *hv* → e^−^ + h^+^

2 2H_2_O + 4h^+^ → 4H^+^ + O_2_

3 2H^+^ + 2e^−^ → H_2_

The entire reaction is

4 2H_2_O + 4*hv* → O_2_ + 2H_2_

Semiconductor photocatalyst materials provide a crucial role in several energy and environmental applications, encompassing processes such as CO_2_ reduction, wastewater degradation, and water splitting.^30^ Nevertheless, the effectiveness of these procedures, especially when utilizing single semiconductors sometimes faces difficulties in attaining both efficient degradation and energy generation.^31^ Due to its narrow bandgap and restricted charge separation, a single semiconductor photocatalyst shows difficulty in rapid electron–hole pair recombination. Over a decade, researchers have focused their efforts on the development of hybrid photocatalysts with the goal of enhancing their light absorption abilities throughout the visible part of the spectrum, hence improving the efficiency of solar energy harvesting process.

## 4. Type of heterojunction photocatalyst

**Fig. 3:**
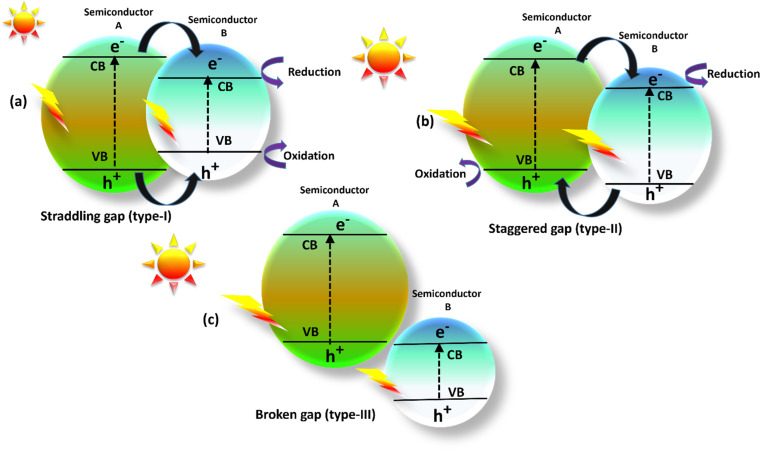
Illustrated heterojunction photocatalyst: (a) straddling gap, (b) staggered gap and (c) broken gap.

The single semiconductor photocatalysts such as TiO_2_,^32^ ZnO,^33^ CuO_2_,^34^ and similar materials, exhibit limitations such as insufficient utilization of visible light, rapid recombination of electron–hole pairs, and wide band gaps. Co-doping techniques effectively address these issues by narrowing band gaps and enhancing charge separation through the incorporation of additional semiconductor materials.^35^ By doping semiconductors, these techniques provide interfaces that facilitate efficient charge transfer and mitigate recombination. Heterojunctions exhibit significant potential to revolutionize photocatalysis for sustainable applications, spanning environmental remediation and energy conversion.^36^ A heterojunction photocatalyst occurs when two semiconductors with different band structures get in connect with each other and may lead to band orientations. According to Fig. 3, there are three different forms of heterojunction photocatalysts: straddling, staggered, and broken junctions.^37^ In the Type-I heterojunction photocatalyst depicted in (Fig. 3a), both photogenerated electrons and holes have the ability to migrate from the semiconductor-A, which possesses a greater conduction band and a lower valence band, to the semiconductor-B. However, due to the Type-I heterojunction configuration, effective charge separation becomes challenging, as electrons and holes tend to transfer within the same semiconductor.^38^ In Type II heterojunctions (Fig. 3b), electrons transfer from the semiconductor-A to a region with a lower CB level of semiconductor-B while holes transfer the semiconductor-B to a higher VB level semiconductor-A. The contact and recombination of electron–hole pairs are reduced.^39^ In the configuration of the Type III heterojunction closely resembles to the Type II heterojunction (Fig. 3c). However, a crucial distinction lies in the fact that the bandgaps of the two semiconductors do not overlap at the interface due to a significant disparity in the energy levels of their respective bands.^40^ The Type II heterojunction exhibits an intrinsic capacity to effectively enhance the separation of electrons and holes, thus exhibiting an attractive option due to the reduced rate of recombination that leads to extended carrier lifetimes. Consequently, Type II heterojunctions are well-suited for applications such as energy and environment.

Yuan *et al.* (2019) reported a novel heterojunction of Type II In_2_S_3_/InVO_4_ photocatalyst which was fabricated by incorporating Na_2_S into a reaction solution containing pre-synthesized InVO_4_ microspheres with In_2_S_3_/InVO_4_ combination as synthesized, exhibited remarkable photocatalytic efficiency in the degradation of tetracycline under the visible light illumination. Remarkable photocatalytic efficiency was observed in the degradation of tetracycline under visible light illumination. The photoactivity of the In_2_S_3_/InVO_4_ composite material exhibit the degradation 11.71 times higher than pristine InVO_4_ and 2.26 times higher than pristine In_2_S_3_. The photocatalytic activity of In_2_S_3_/InVO_4_ was highly improved due to the photosensitization of InVO_4_ by In_2_S_3_ and enhancement of charge separation *via* the compact interface junction between the semiconductors.^41^ Huang *et al.* had also fabricated various hierarchical heterojunctions such as g-C_3_N_4_/BiOI (Type I heterojunction), g-C_3_N_4_/Bi_4_O_5_I_2_ (Type I heterojunction), and g-C_3_N_4_/Bi_5_O_7_I (Type II heterojunction). These were achieved using processes involving direct precipitation and *in situ* calcination transformation. The g-C_3_N_4_/Bi_5_O_7_I composite demonstrated exceptional photodegradation activity towards micropollutants such as tetracycline and rhodamine-B. These can be attributed to its enhanced specific surface area, which facilitates effective charge separation in the Type II heterojunction.^42^

## 5. Photocatalytic degradation of micropollutants

This review provides an in-depth exploration of micropollutants degradation *via* photocatalysis. Our focus lies on micropollutants such as pharmaceutical compounds, phenolic compounds, organic dyes, pesticides, petroleum refinery effluent, and other industrial chemicals. By delving into these harmful substances, we aim to provide a comprehensive understanding of their environmental and industrial implications. Organic pollutants share numerous concerning characteristics with heavy metal contamination such as Cr(vi).^43^ These toxins are non-biodegradable and persist in the environment for prolonged periods. Due to their tendency to accumulate and cause gradual harm, persistence poses a significant concern. Micropollutants typically pose hazards, posing risks to both human health and the environment. Their toxicity has the potential to disrupt ecosystems and yield long-lasting consequences.^44^

Furthermore, these pollutants exhibit resistance to degradation. Unlike other contaminants, these organic toxins are challenging to break down or remediate. This resistance contributes to their extended lifespan. This review has been conducted on various aspects, including removal strategies, occurrence patterns, sources, and the fate of these substances. The upcoming sections will delve into the multiple facets of this subject. Through this review, we present a thorough examination, aiming to enhance the understanding of micropollutants and their intricate behaviours.

### 5.1. The impact of pharmaceuticals wastewater on ecosystems

The most commonly used antibiotics and medications are applied for human, agricultural, and veterinary treatments, among the other applications. On a yearly basis, a massive amount of antibiotics and medications are consumed around the world. Recently, pharmaceutical waste products in the milligram per litre range have been recognized in various sources of water such as drinking water, groundwater, sewage from municipalities, and furthermore. Pharmaceutical pollutants contain unidentified reactive pollutants that have a variety of negative effects on the wellness of people, aquatic life, and the environment.^26^ Even in modest amounts, these environmental contaminants are capable of causing a variety of negative consequences, including chronic toxicity, disturbance of the endocrine system, and resistance to antibiotics.^45–47^

### 5.2. Single semiconductor photocatalyst

**Fig. 4:**
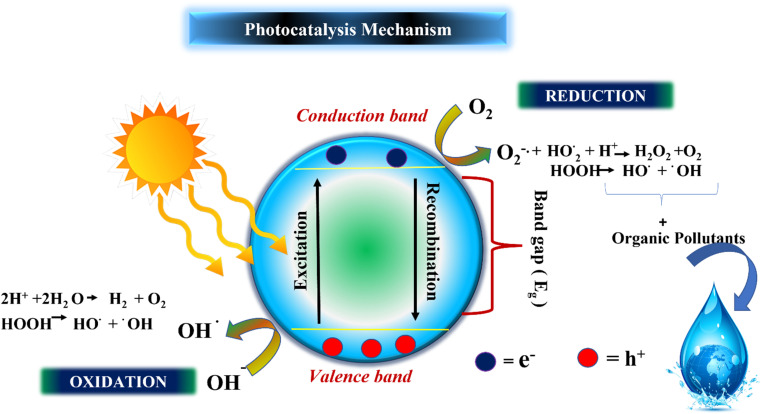
Schematic diagram represented a single semiconductor photocatalytic degradation mechanism.

The ideal photocatalyst to promote the complete mineralization of antibiotics must have several key attributes. These include low toxicity, making it safe for use in various applications. Affordability is also important, ensuring that the photocatalyst is accessible to a wide range of users. Stability is another crucial factor, as it ensures the longevity and effectiveness of the photocatalyst over time. Additionally, accessibility is essential, allowing for easy obtaining and utilization. Moreover, strong photoactivity is an essential characteristic, enabling efficient and effective mineralization of antibiotics. Single-component photocatalysts have a certain number of advantages, however, it's important to recognize some inherent disadvantages as well.^48^ Moreover, single-component photocatalysts such as ZnO, SnO_2_, WO_3_, and TiO_2_ offer numerous advantages, including their non-toxic nature, cost-effectiveness, and strong stability (Fig. 4). However, due to their wide bandgap, these materials are restricted to functioning as photocatalysts primarily in the ultraviolet spectrum, as we discussed earlier. Conversely, BiVO_4_ possesses a narrow bandgap, enabling it to participate in the breakdown of antibiotics using visible light. Nevertheless, its photoexcited carriers have a limited lifespan, which could potentially reduce its overall photocatalytic efficiency.^49,50^ Ag_3_PO_4_, a semiconductor photocatalyst, demonstrates impressive efficiency in the degradation of antibiotics when exposed to visible light. However, the practical application of Ag_3_PO_4_ is hindered by its restricted stability. The excess electrons in the Ag_3_PO_4_ conduction band led to significant self-photo corrosion. In addition, Ag_3_PO_4_ has moderate water solubility (0.00065 g/100 mL).^51^

The majority of single-component photocatalysts exhibit remarkable effectiveness in removing antibiotics from water when exposed to visible-light radiation. However, previous research has either neglected the mineralization process or reported low levels of total organic carbon reduction. For instance, a study indicated that Bi_2_WO_6_ achieved complete tetracycline removal of 100% after 360 minutes of ultraviolet-visible-light exposure. Yet, it's crucial to highlight that the elimination of total organic carbon through this process only reached a modest 31%. This observation suggests that the degradation of tetracycline primarily leads to the creation of intermediate compounds rather than complete degradation.^52^ The integration of hybrid photocatalytic systems comprising multiple components holds immense potential for the degradation of antibiotics. By harnessing the synergistic contributions of each component within the system, this approach enhances antibiotic degradation. The adoption of multicomponent photocatalysts has resulted in notable enhancements in their photocatalytic efficiency. Additionally, these systems have showcased an impressive capability to achieve substantial levels of mineralization.

### 5.3. Two-component hybrid semiconductor photocatalyst

**Fig. 5:**
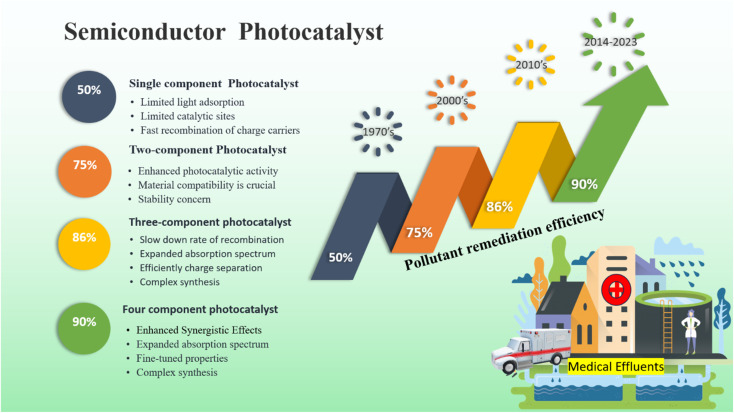
Represented the pollutant remediation efficiency by semiconductor heterojunction photocatalysts.

Recently, the development of heterojunction materials has been considered a potential technique to improve the effectiveness of photocatalytic processes.^53^ The studies are mainly focused on the synthesis of heterojunction photocatalysts used in the process of photocatalytic degradation of antibiotics, and classification of semiconductor photocatalyst as shown in Fig. 5. In general, three primary approaches have been employed in the development of photocatalysts, which involve the incorporation of (1) semiconductor–metal; (2) semiconductor–adsorbent, and (3) semiconductor–semiconductor heterojunctions. This section will provide a discussion on the degradation mechanisms of antibiotics using heterojunction photocatalysts.

#### 5.3.1. Semiconductor–metal photocatalyst

The construction of metals with semiconductors to create semiconductor–metal heterojunction photocatalysts has been the focus of extensive research endeavours. Several research studies have successfully employed this approach to harness the benefits of photocatalytic degradation of antibiotics. This method generally presents two primary benefits that render it highly attractive. Firstly, it possesses a prolonged carrier lifetime, which confers significant advantages. Additionally, it offers improved absorption of visible light, so augmenting its attractiveness.^54^ It is worth noting that the semiconductor–metal heterojunction is a prevalent characteristic. In general, the composition of this material comprises an n-type semiconductor, specifically TiO_2_, in conjunction with noble metals such as Cu, Bi, Au, Pt, Pd, and Ag. The surface interaction between this semiconductor and metal component is when the transformative process occurs. An intriguing phenomenon arises as a result of the metal possessing a work function that is higher than the conduction band position of the n-type semiconductor. Fermi energy levels are aligned as electrons travel effectively from the n-type semiconductor to the noble metal nanoparticles. The presence of an excessive number of negative charges on the metal nanoparticles and an excessive number of positive charges on the semiconductor leads to the formation of a Schottky barrier. The improved photocatalytic efficacy can be ascribed to the efficient capture of conduction band electrons at the Schottky barrier, hence reducing the occurrence of recombination among the photoinduced charge carriers.^55,56^ Luo *et al.*^57^ conducted investigations to determine the efficient photocatalytic activity of Ag/Bi_3_TaO_7_, when provided with sufficient energy, the excited electron (e^−^) in the valence band of Bi_3_TaO_7_ moves to the conduction band of Bi_3_TaO_7_. When provided with sufficient energy, the excited electron (e^−^) in the valence band of Bi_3_TaO_7_ moves to the conduction band of Bi_3_TaO_7_. This process leaves behind a positively charged hole (h^+^) in the valence band of Bi_3_TaO_7_, as shown in (eqn (5)). The energy level of the valence band in Bi_3_TaO_7_ is higher than the redox potentials of –OH/˙OH (1.99 V *vs.* NHE) and H_2_O/˙OH (2.7 V *vs.* NHE). The photocatalyst reacts with light to separate water molecules into hydrogen (H^+^) and hydroxide (OH^−^) ions (eqn (6)). As a result, the photogenerated h^+^ species directly react with –OH/H_2_O, leading to the generation of ˙OH radicals, as depicted in (eqn (7)). Concurrently, the metallic silver nanoparticles that have been arranged on the surface of bismuth tantalate (Bi_3_TaO_7_) also exhibit photoactivation due to the generation of electron–hole (e^−^/h^+^) carriers generated by plasmon effects. The conduction band level of Bi_3_TaO_7_ (0.32 V *vs.* NHE) is observed to be situated at a lower level of energy compared to the Fermi position of Ag (0.4 V *vs.* NHE). As a result, the photogenerated electrons in the valence band of Bi_3_TaO_7_ have the potential to be obtained by the plasmon-induced holes in the metallic Ag nanoparticles (as described in (eqn (8))). It is worth noting that the plasmon-induced electrons in the metallic Ag can diffuse towards the Ag surface and subsequently react with O_2_ in water, resulting in the formation of ˙O_2_^−^ active species as depicted in (eqn (9)) The active species, like ˙O_2_^−^ and ˙OH, have the capability to undergo oxidation of tetracycline, resulting in the formation of intermediates or final products such as carbon dioxide and water molecules as described in (eqn (10)).

5 Bi_3_TaO_7_ + *hv* → Bi_3_TaO_7_(h^+^) + Bi_3_TaO_7_(e^−^)

6 H_2_O → H^+^ + OH^−^

7 Bi_3_TaO_7_(h^+^) + OH^−^ → HO˙

8 Bi_3_TaO_7_(e^−^) + Ag → Ag(e^−^)

9 Ag(e^−^) + O_2_ → ˙O_2_^−^

10
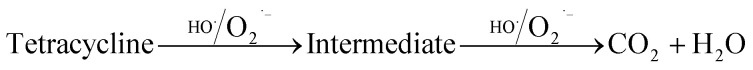


The increased degradation of antibiotics achieved with Ag/Bi_3_TaO_7_ can be attributed to the incorporation of Ag nanoparticles onto the surface of Bi_3_TaO_7_. The role of these Ag nanoparticles is to function as electron traps during the degradation process. This, in turn, effectively extends the lifetime of the photogenerated carriers. Additionally, the introduction of Ag nanoparticles within the system acts as photosensitizers, capitalizing on the surface plasmon resonance effect. Consequently, this outcome leads to a broadened absorption spectrum of visible light, in contrast to the behavior observed with bare Bi_3_TaO_7_.^57^ Similarly, the incorporation of Ag in the Ag/WO_3_ nanoplates visible-light system proved to be highly effective in trapping electrons and acting as an organic photosensitizer. This resulted in a significant improvement in the photocatalytic degradation of sulfanilamide. After 5 h, the Ag/WO_3_ nanoplates achieved an incredible degradation efficiency of 96.1% and a total organic carbon removal rate was 73.4%.^58^

Petronella *et al.* (2013) reported a study on the degradation of nalidixic acid using TiO_2_ nanorods/Ag nanoparticles under UV radiation.^59^ The study attributed the observed improvement in degradation in to two primary factors: the efficient separation of electron–hole pairs (e^−^/h^+^) and the significant surface area of the TiO_2_ nanorods/Ag nanoparticles. However, it is important to note that the study did not explore the enhanced absorption of visible light. The observed phenomenon can be associated with the surface plasmon resonance phenomenon exhibited by noble metals, which is dependent upon the size, morphology, and arrangement.^60^ Ag-based composite photocatalysts, specifically Ag/TiO_2_, have been demonstrated with enhanced efficiency in the photocatalytic degradation of antibiotics,^61^ which include Ag/K_2_Ta_2_O_6_,^62^ Ag/BiO_4_Cl,^63^ Ag/BiOBr,^64^ and Ag/Bi_2_WO_6_.^65^

The incorporation of a bimetallic semiconductor into its design, along with the development of two different kinds of novel metal nanoparticles, aims to develop materials that possess enhanced mineralization and degradation efficiency.^66^ The photocatalytic degradation rate of levofloxacin can be significantly enhanced through the utilization of the Au–Pd/TiO_2_ combination. This enhancement results in an impressive degradation efficiency of approximately 95% within 1 h of exposure to simulated solar light. Furthermore, under visible light exposure, the degradation efficiency reaches around 93% within 1.67 h. These results unequivocally demonstrate the superior performance of Au–Pd/TiO_2_ compared to unmodified TiO_2_, as well as the individual metal–semiconductor systems of Au/TiO_2_ and Pd/TiO_2_.^67^ Besides this, mineralization occurred, which led to the elimination of almost 90% of total organic carbon during a duration of 1 h upon exposure to simulated solar radiation. The enhanced degradation of antibiotics observed on a bimetallic Au–Pd co-anchored TiO_2_ surface can be attributed to three factors: (1) the incorporation of a bimetallic Au–Pd catalyst effectively captures electrons that are generated during photocatalytic reactions, thereby preventing the recombination process of photogenerated charge carriers. (2) The bimetallic Au–Pd co-anchored TiO_2_ surface demonstrates superior performance compared to a single metallic Au-anchored TiO_2_ surface in the production of H_2_O_2_, which is a crucial active species in the photocatalytic reaction. (3) The presence of bimetallic Au–Pd enhances the utilization efficiency of photoexcited electrons and oxygen, resulting in a higher quantum efficiency for Au–Pd/TiO_2_ compared to Au/TiO_2_.

#### 5.3.2. Dual-semiconductor photocatalyst

To address the issues of fast recombination of charge carrier as well as poor reduction and oxidation potential in single component semiconductors, the development of semiconductor–semiconductor heterojunction photocatalyst with employing a bandgap and band energy that are appropriate is a feasible strategy. On the process of photocatalytic degradation of pharmaceutical waste water and antibiotics, five systems have been extensively studied Type-II, p–n, n–n, Z-scheme, and S-scheme heterojunctions. This addresses the difference in charge transfer processes of these heterojunction in antibiotic photocatalytic degradation. The development of Type-II semiconductor heterojunctions has emerged as a promising strategy for enhancing the photocatalytic performance of semiconductor–semiconductor photocatalyst materials. By effectively separating electron (e^−^) and hole (h^+^) carriers, these heterojunctions provide a valuable manner for improving the efficiency of photocatalysis. Among the various Type-II heterojunction photocatalysts that have been investigated, the synthesis of p–n heterojunction photocatalysts, facilitating the effective transfer of positive (h^+^) and negative (e^−^) charge carriers between p-type and n-type semiconductors, which has led to the development of materials with enhanced photocatalytic activity. An investigation carried out by Wen *et al.* reported the use of a BiOI/SnO_2_ composite photocatalyst to enhance the degradation process of oxy-tetracycline under visible light conditions.^68^ The energy band diagram illustrates the potential formation of a p–n junction at the interface of BiOI and SnO_2_ within the BiOI/SnO_2_ heterojunction. Upon contact between BiOI and SnO_2_, an inner electric field is established due to the consistent alignment of their interaction energy band structures. This built-in electric field facilitates the migration of excited electrons (e^−^) and holes (h^+^) in opposing directions.

Under sufficient photon exposure, the BiOI/SnO_2_ surface undergoes a process where electrons (e^−^) in the valence band of BiOI become excited and subsequently transitioned to the conduction band of BiOI. This transition generates positively charged holes (h^+^) in the valence band. Following this, the excited electron within the valence band of bismuth oxy-iodide (BiOI) preferentially diffuses towards the conduction band of tin dioxide (SnO_2_), aided by the internal electric field. The h^+^ species situated in the valence band of SnO_2_ contribute to the direct oxidation of pollutants. Conversely, the e^−^ species located in the conduction band of BiOI combine with surface-absorbed O_2_ to produce ˙O^2−^ species. These ˙O^2−^ species play a crucial role in facilitating the photocatalytic degradation process by providing additional active species.^68^

**Fig. 6:**
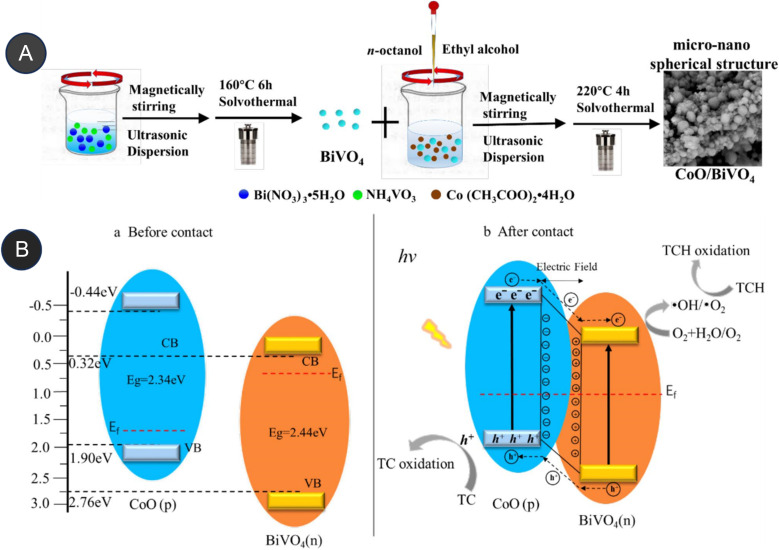
(A) Synthesis of micro-nano-spherical shaped CoO/BiVO_4_, (B) proposed mechanism of p–n heterojunction, CoO/BiVO_4_ photocatalyst before contact and after conduct. Reproduced with permission from ref. 70. Copyright 2021. Elsevier.

Similarly, Sun *et al.* (2020) investigated the fabrication of an Ag_3_VO_4_/Ag_2_CO_3_ p–n heterojunction photocatalyst synthesis by using a one-step coprecipitation method.^69^ The p–n heterojunction photocatalyst significantly enhances the separation and endurance of photogenerated electrons (e^−^) and holes (h^+^), which led to enhanced photocatalytic degradation of antibiotics when exposed to irradiation under a xenon lamp (500 W) with a UV filter, Ag_3_VO_4_/Ag_2_CO_3_ had more effective degradation efficiencies than bare Ag_3_VO_4_ and Ag_2_CO_3_ alone after 60 minutes of irradiation of levofloxacin and tetracycline at almost 82% and 75.1%, respectively. After 60 minutes of irradiation, the amount of total organic carbon elimination likewise achieves a significant value of around 70%.^69^ Wang *et al.* reported the fabrication of p–n heterojunctions between CoO and BiVO_4_, which are noble-metal-free materials, was achieved using a solvothermal method. The resulting heterojunctions CoO/BiVO_4_ exhibited a micro-nano-spherical shape, as seen in Fig. 6. The combination of p-type CoO and n-type BiVO_4_ led to the formation of a p–n junction, facilitating the separation and lifetime of photogenerated electrons (e^−^) and hole (h^+^) carriers. It exhibited a reported quantum efficiency of 0.54%. Compared to bare CoO and BiVO_4_, this CoO/BiVO_4_ composite has enhanced photocatalytic activity in the decomposition and mineralization of tetracycline. The total organic carbon removal, chemical oxygen demand, and photocatalytic degradation efficiency of the composite were determined as 73.8%, 72.1%, and 87.3%, respectively.^70^ Two-component photocatalysts based on Type-II or p–n heterojunction semiconductors have been demonstrated to enhance the separation and migration of photoexcited e^−^/h^+^ pairs. However, their redox capabilities are limited by the constrained reduction and oxidation potentials of their components. These challenges can be addressed through the construction of Z-scheme heterojunction photocatalysts.

In the case where a Z-scheme heterojunction photocatalyst comprises semiconductors A and B and is exposed to light irradiation, both semiconductor components become excited, resulting in the generation of photoexcited electrons (e^−^) and holes (h^+^). Specifically, the photoexcited electron located in the conduction band of semiconductor A, possessing a lower reduction potential, exhibits a propensity to migrate towards the valence band of semiconductor B, which has a lower oxidation potential. This behaviour is driven by the electrostatic attraction between the electron and the positively charged holes (h^+^). Moreover, the electrons generated by the photochemical process that remain in semiconductor B, as well as the holes left in semiconductor A, have the potential to engage with species such as OH^−^, H_2_O, and/or O_2_, leading to the creation of active radicals. This phenomenon is attributed to their heightened reduction and oxidation potentials, respectively. Specifically, the photoexcited electron located in the conduction band of semiconductor A, possessing a lower reduction potential, exhibits a propensity to migrate towards the valence band of semiconductor B, which has a lower oxidation potential. This behaviour is driven by the electrostatic attraction between the electron and the positively charged holes (h^+^). Moreover, the electrons generated by the photochemical process that remain in semiconductor B, as well as the holes left in semiconductor A, have the potential to engage with species such as OH^−^, H_2_O, and/or O_2_, leading to the creation of active radicals. This phenomenon is attributed to their heightened reduction and oxidation potentials, respectively.

**Fig. 7:**
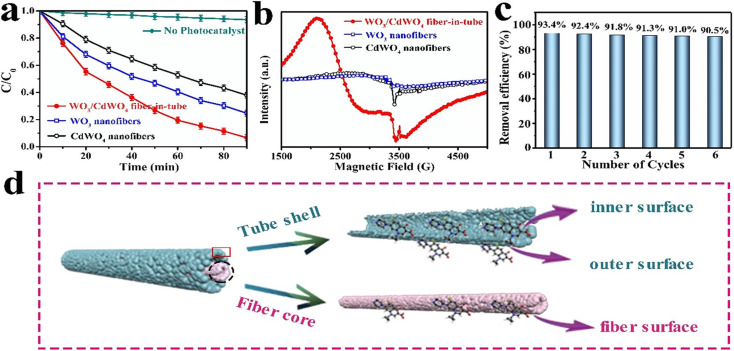
(a) Photocatalytic degradation of ciprofloxacin, (b) EPR spectra of WO_3_/CdWO_4_, WO_3_, and CdWO_4_, (c) photocatalytic degradation efficiency of six cycles and (d) fabrication of FITNs. Reproduced with permission from ref. 71. Copyrights 2021, American chemical society.

Similarly, Rong *et al.* investigated the electrospinning method to fabricate fiber-in-tube nanostructures (FITNs) composed of WO_3_/CdWO_4_, featuring a hierarchical porosity structure.^71^ These composite materials were designed with a Z-scheme heterojunction configuration, which facilitated the generation of superoxide anions and hydroxyl radicals. Consequently, when subjected to photocatalytic degradations in aqueous solutions, these materials effectively catalysed the degradation of antibiotics, particularly ciprofloxacin (CIP) and tetracycline (TC). Notably, the novel heterostructure exhibited a substantial enhancement in its photocatalytic performance for the degradation of CIP and TC, achieving degradation rates of 93.4% and 81.6%, respectively, following a 90 min exposure to sunlight irradiation. Comparatively, the degradation rates attained by the individual pristine materials WO_3_ and CdWO_4_ were notably lower than the values reported in this study (Fig. 7). Specifically, the degradation rates for WO_3_ were less than 75.3% for CIP and less than 53.6% for TC.^71^

#### 5.3.3. Semiconductor-adsorbent photocatalyst

The designing of heterojunction photocatalysts using the combination of a semiconductor and an adsorbent photocatalyst is another method for photocatalytic degradation, that can be used to improve the photocatalytic performance of photocatalysts. Adsorbents with a large specific surface area and functional groups have the potential to provide improvements in photocatalytic reactions due to their ability to present a considerable quantity of surface-active sites. Consequently, it is possible to synthesize compounds that demonstrate a significant photocatalytic degradation rate, low toxicity, and a high degree of efficiency in the mineralization of organic compounds.^72^

For example, the reduced graphene oxide (RGO)–WO_3_ composite exhibited exceeded performance in the photocatalytic degradation of sulfamethoxazole. This resulted in a photocatalytic degradation rate of 98% and a removal of total organic carbon of 64.49% at the 180 min time period under visible light illumination. The enhancement of this composite material is emphasized by few main characteristics. The RGO is widely recognized for its extensive surface area and rich functional groups, thus providing an excellent carrier that provides several adsorption and catalytic sites.^73^ As a result, this phenomenon enhances the photocatalytic activity. Furthermore, the RGO efficiently absorbs migrating electrons (e^−^) generated by WO_3_, thereby preventing the recombination of the charge carriers.

This can be elucidated by examining the envisaged mechanism of charge transfer within the RGO/WO_3_ composite. Following exposure to visible light, the RGO matrix adeptly captures photoinduced electrons (e^−^) diffusing from WO_3_. The observed occurrence can be attributed to the relatively low Fermi level of RGO (EF = −4.26 eV) and its strong electrical conductivity. Following this, the accumulated electrons inside the matrix of RGO interact with molecular oxygen (O_2_), resulting in the production of superoxide radicals (˙O_2_^−^). Subsequently, these radicals interact with further electrons (e^−^) and/or holes (h^+^) species, resulting in the production of extremely reactive hydroxyl radicals (OH˙). Furthermore, the residual positive holes (h^+^) in WO_3_ engage with –OH or H_2_O, resulting in the precipitation of ˙OH radicals. Essentially, the ˙OH radicals function as highly effective oxidizing agents, demonstrating their effectiveness in breaking down sulfamethoxazole molecules, thereby contributing to the remarkable photocatalytic ability of the composite material.^74^

### 5.4. Three-component hybrid photocatalyst

In recent decades, there has been a development of multicomponent photocatalysts comprising three or four components. These photocatalysts are intended for applications in the degradation of antibiotics. Several concepts have been documented in the literature, including the synthesis of metal/p–n heterojunctions, metal/semiconductor/absorbent systems, metal/Z-scheme heterojunctions, and p–n heterojunction/absorbent photocatalyst systems. These systems employ the synergistic effects of their components to create novel photocatalysts that offer various advantages, such as an expanded light absorption spectrum, enhanced charge transfer efficiency, and fine-tuned properties.^75^

**Fig. 8:**
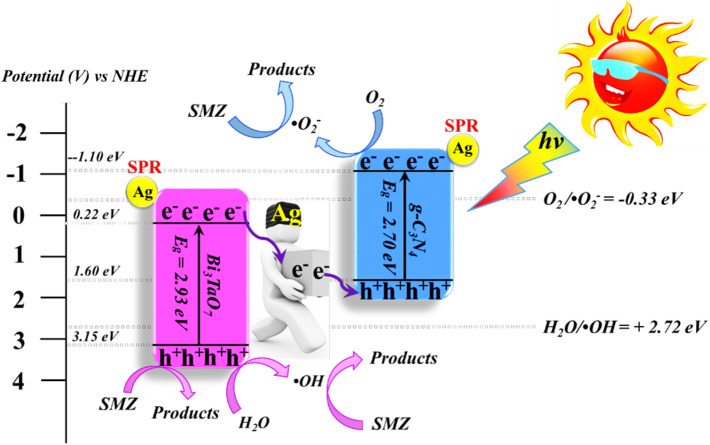
Proposed mechanism for photocatalytic degradation of sulfamethoxazole by Ag/g-C_3_N_4_/Bi_3_TaO_7_ ternary composite photocatalyst. Reproduced with permission ref. 76, Copyright 2019.

Metallic silver nanoparticles (Ag) have found extensive use in enhancing visible light absorption and promoting charge transfer in photocatalysts containing metal/semiconductor heterojunctions. This is primarily due to their capability to induce a surface plasmon resonance effect. In a study conducted by Ren *et al.* (2019), they demonstrated that ternary composites of Ag/g-C_3_N_4_/Bi_3_TaO_7_ exhibited enhanced electron–hole transfer and broader visible-light responsiveness. This enhancement was attributed to the dual functionality of silver, which acted as both a mediator and a photosensitizer. The outcomes of this research are illustrated in Fig. 8. Moreover, the presence of a higher valence band level in Bi_3_TaO_7_ and a lower conduction band level in g-C_3_N_4_ contribute a significant role in enhancing photocatalytic activity. Consequently, the Ag/g-C_3_N_4_/Bi_3_TaO_7_ ternary composites displayed superior photocatalytic performance in sulfamethoxazole degradation. Their performance was approximately 83.3 times better than bare Bi_3_TaO_7_, 5.6 times better than g-C_3_N_4_, and 2.2 times better than the binary composite g-C_3_N_4_/Bi_3_TaO_7_,^76^ Ag/AgBr/BiVO_4_,^77^ and BiVO_4_/Ag/Cu_2_O,^78^ which efficiently degraded. Similar reports have been investigated by Li *et al.*^79^ The fabrication of a novel 3D composite of Ag/Ag_6_Si_2_O_7_/Bi_2_MoO_6_ trible component photocatalysts were synthesized hydrothermally and the degradation performance of the photocatalyst have been studied efficiently with the antibiotic such as ciprofloxacin and tetracycline hydrochloride. The proposed mechanism elucidates the charge transfer pathways within a triple-junction Ag/Ag_6_Si_2_O_7_/Bi_2_MoO_6_ heterojunction photocatalyst system, offering a comprehensive understanding of the combined impacts of the Ag_6_Si_2_O_7_/Bi_2_MoO_6_ p–n heterojunction and the plasmonic influence of Ag. These factors synergistically account for the notable enhancement in photocatalytic activity within this three-component system. The interaction of charge transfer occurs between the p-type semiconductor Ag_6_Si_2_O_7_ and the n-type semiconductor Bi_2_MoO_6_, playing a pivotal role in restricting the recombination of photogenerated electron and hole pairs. Moreover, the inclusion of Ag nanoparticles expediates the charge transfer process, effectively curtailing the recombination of these electron–hole pairs. Similarly, it has been demonstrated that a triple-component system consisting of Ag/AgCl@In_2_O_3_ exhibits remarkable photocatalytic performance. Specifically, this system achieves a photodegradation rate of 99.5% for tetracycline antibiotics within a 30 min period of irradiation using a 300 W Xenon lamp that is equipped with a UV filter.^80^ In the ternary composite of Ag/AgCl@In_2_O_3_, there exists a lack of charge transfer pathways between AgCl and In_2_O_3_. The generation of e^−^ in the Ag particles is a result of the surface plasmon resonance effect, which then migrates to the conduction band of In_2_O_3_ and/or AgCl. Furthermore, the photogenerated h^+^ in the Ag particles combines with the photogenerated e^−^ in AgCl. The accumulated e^−^ in the conduction band of In_2_O_3_ reacts with O_2_ in water, leading to the production of ˙O_2_^−^. The h^+^ in In_2_O_3_ directly interacts with tetracycline, facilitating its degradation. In parallel, the h^+^ in AgCl oxidizes Cl^−^ to Cl˙. It is likely that the active species ˙O_2_^−^, h^+^, and Cl˙ collectively contribute to the photocatalytic degradation of tetracycline. Chen *et al.* reported a study on a Bi_2_O_3_/(BiO)_2_CO_3_ composite material's heterojunction for the photocatalytic degradation of the antibiotic CIP under visible light exposure. The outcomes showed that the Bi_2_O_3_/(BiO)_2_CO_3_ composite exhibited a remarkable enhancement in degradation efficiency. This improvement was quantified through ultraperformance liquid chromatography coupled with a mass analyzer (UPLC/MS), revealing a degradation rate of 93.4% for ciprofloxacin within a 30 minutes timeframe.^81^ Influence of visible light, metallic silver (Ag) accumulated and acted as a mediator for electron transfer, facilitating the establishment of a dual Z-scheme heterojunction by linking recombined electrons (e^−^) and holes (h^+^) were described. Consequently, the photocatalyst synthesized in this investigation, comprising of Ag_2_CO_3_, CeO_2_, and AgBr. The degradation of levofloxacin was observed under the influence of a 300 W Xenon lamp fitted with a 420 nm cutoff filter. The composite material consisting of Ag_2_CO_3_/CeO_2_/AgBr which exhibited an impressive degradation efficiency of 87.63% within 40 min. In comparison, the degradation efficiencies of Ag_2_CO_3_/CeO_2_, Ag_2_CO_3_, and CeO_2_ were approximately 70%, 50%, and 0%, respectively, over a 60 min period. The Ag_2_CO_3_/CeO_2_/AgBr catalyst demonstrated remarkable efficacy in the mineralization of levofloxacin, resulting in a substantial 60.98% reduction in total organic carbon after 80 minutes of irradiation. Furthermore, following the photocatalytic reaction using Ag_2_CO_3_/CeO_2_/AgBr, the toxicity of the reaction solution was effectively mitigated due to the inherent biodegradability of the reaction solution. This was evidenced by a notable increase in the BOD and COD values from 0.096 to 0.691. The byproducts generated during the photocatalytic degradation of levofloxacin using the Ag_2_CO_3_/CeO_2_/AgBr composite could potentially be efficient and can be safely removed.^82^

### 5.5. Four component hybrid photocatalyst

The process of charge separation is of crucial significance in enhancing the photocatalytic efficiency of semiconductor materials. In the case of quaternary photocatalytic systems, the migration of charge carriers occurs through several steps, which subsequently promotes the separation of charges and restricted the recombination rate.^83^ As a consequence, these systems demonstrate enhanced photocatalytic efficiency compared to single, binary, and ternary systems. Several four-component photocatalysts exhibiting multiple proposed mechanisms for the photocatalytic degradation of antibiotics have been reported such as g-C_3_N_4_/Ag/AgCl/BiVO_4_,^84^ Ag–AgCl/WO_3_/g-C_3_N_4_,^85^ Ag/AgBr/TiO_2_/RGO,^86^ and Cu_2_O-CdS-BiVO_4_–WO_3_.^87^

Fan *et al.* (2021) investigated the very excellent active g-C_3_N_4_ modified with WO_3_ and Ag–AgCl. The Ag–AgCl/WO_3_/g-C_3_N_4_ composite exhibits a remarkable photodegradation rate towards trimethoprim following a 90 min exposure to visible light. When considering the individual components WO_3_ and g-C_3_N_4_, it is observed that the binary junction catalyst WO_3_/g-C_3_N_4_, as well as the ternary junction catalysts Ag–AgCl/WO_3_ and Ag–AgCl/g-C_3_N_4_, exhibit notable differences. The presence of a dual charge transfer configuration has proposed in the four-component photocatalyst. This configuration demonstrates enhanced degradation of trimethoprim when exposed to visible light. It involves a direct Z-scheme charge transfer mechanism between WO_3_ and g-C_3_N_4_, as well as the inclusion of plasmonic Ag as a mediator for charge transfer between the conduction bands of AgCl and g-C_3_N_4_.^85^ The photocatalytic efficiency of a quaternary junction Ag–AgBr/TiO_2_/RGO photocatalyst has been demonstrated more effective over the performance of TiO_2_ and various heterojunction composites, including Ag–AgBr, Ag/TiO_2_, TiO_2_/RGO, Ag–AgBr/RGO, Ag–AgBr/TiO_2_, and Ag/TiO_2_/RGO systems, for the degradation of penicillin-G under white light-emitting diode illumination. This remarkable result observed in the study can be attributed to the combined influence of each constituent in the four-component catalyst. These components include; (1) the surface plasmon resonance effect exhibited by Ag nanoparticles. (2) The enhanced visible-light absorption facilitated by the high visible-light reactivity of AgBr. (3) The sequential movement of charge carriers between Ag–AgBr, TiO_2_, and RGO. (4) The transfer of charge carriers along the π–π graphitic carbon network at the interface.^86^ In their 2018 study, Kumar *et al.* discovered a quadripartite composite photocatalyst composed of BiOCl/g-C_3_N_4_/Cu_2_O/Fe_3_O_4_, which exhibited impressive photodegradation and mineralization rates for sulfamethoxazole (SME). In comparison to triple-junction systems (C_3_N_4_/BiOCl/Fe_3_O_4_, Cu_2_O/BiOCl/Fe_3_O_4_, and Cu_2_O/BiOCl/C_3_N_4_), this quadripartite photocatalyst displayed exceptionally high photocatalytic efficiency. It achieved a remarkable 99.5% degradation of SME within just 1 h of exposure to visible light and 92.1% degradation within 2 h of exposure to natural sunlight. Furthermore, this reaction system eliminated 41.6% of the total organic carbon after 3 h of visible light exposure. To confirm the effectiveness of antibiotic degradation, bactericidal activity tests were conducted against *E. coli*, and cytotoxicity assessments were performed on human peripheral blood cells. In the bactericidal activity tests, the inhibition zones measured 2.1 nm and 18.1 nm following exposure to pure SME and photo-catalytically degraded SME, respectively, indicating complete mineralization of the antibiotic. In terms of cytotoxicity, human peripheral blood lymphocyte cells treated with photocatalytically-degraded SME exhibited 99% cell viability. The exceptional photodegradation and mineralization rates of SME by the four-component BiOCl/gC_3_N_4_/Cu_2_O/Fe_3_O_4_ catalyst were attributed to an accelerated interfacial charge transfer process facilitated by the inherent electric field (n–p–n junction). Moreover, the catalyst's magnetic characteristics, stemming from the Fe_3_O_4_ component, contributed to its high reusability, making it a versatile and efficient catalyst for degrading pharmaceuticals in water.^88^Table 1 summarizes several semiconductor heterojunctions used in the photocatalytic degradation of pharmaceutical compounds.

**Table 1:** Photocatalytic degradation of various pharmaceutical compounds

S. no.	Photocatalyst	Catalyst mass	Micropollutant	Pollutant mass	Light source	Removal efficiency	Time (hour)	Ref.
1	Ag TiO_2_	8 mg	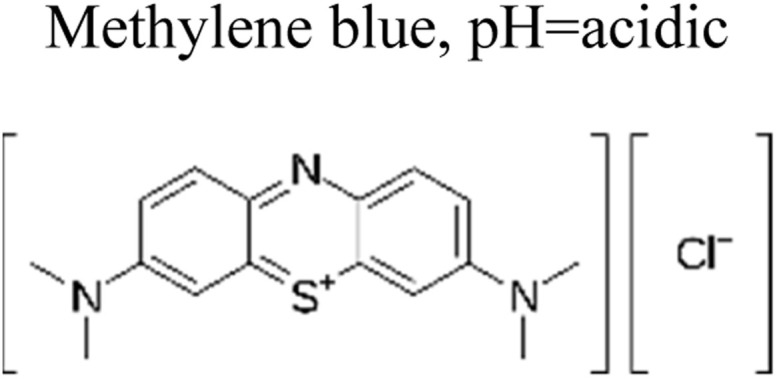	5 mg L^−1^	Halogen lamp (300 W)	90%	0.66 h	167
2	g-C_3_N_4_–Ag–TiO_2_	1000 mg	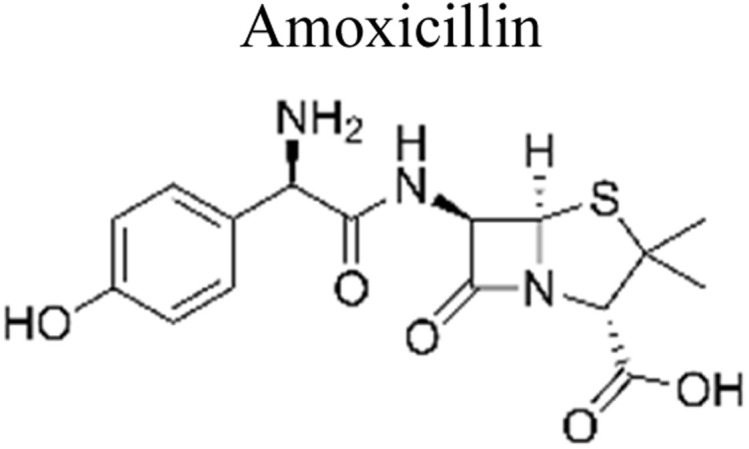	20 mg L^−1^	Tungsten–halogen lamp (500 W)	73.4%	6 h	168
3	Au@TiO_2_ bio-based chitosan fiber	5 mg	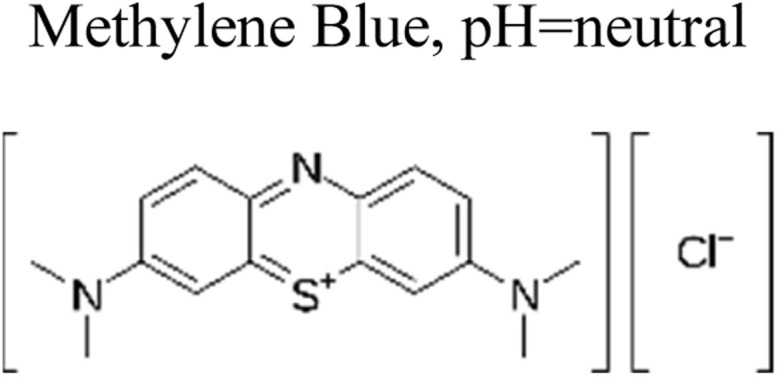	10 mg L^−1^	Halogen lamp (250 W)	98.8%	0.58 h	169
4	CuInS_2_/ZnS	40 mg	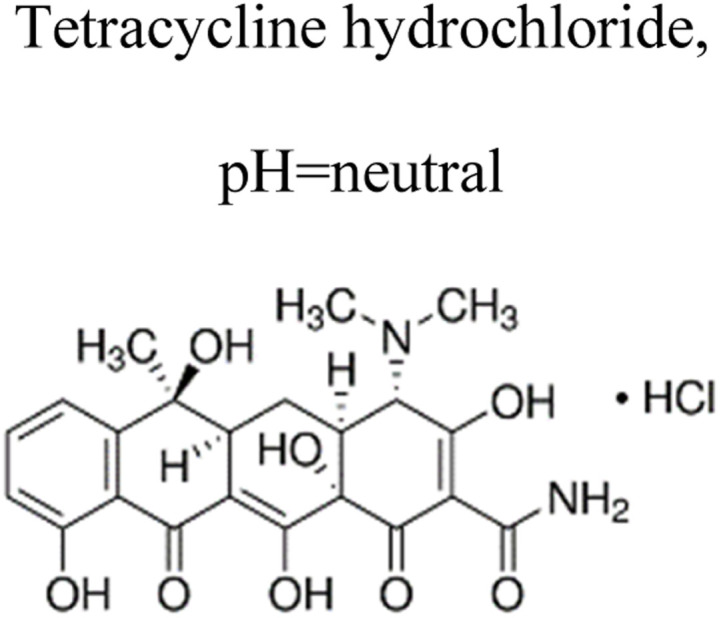	60 mg L^−1^	Xenon lamp (300 W)	86%	3 h	170
5	Ag/ZnNb_2_O_6_@SC_3_N_4_	40 mg	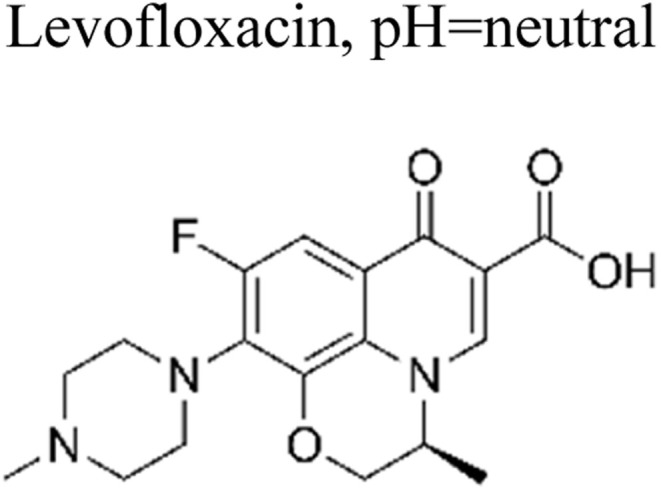	10 mg L^−1^	Tungsten lamp (300 W)	94.6%	1 h	171
6	CuO–NiO	80 mg	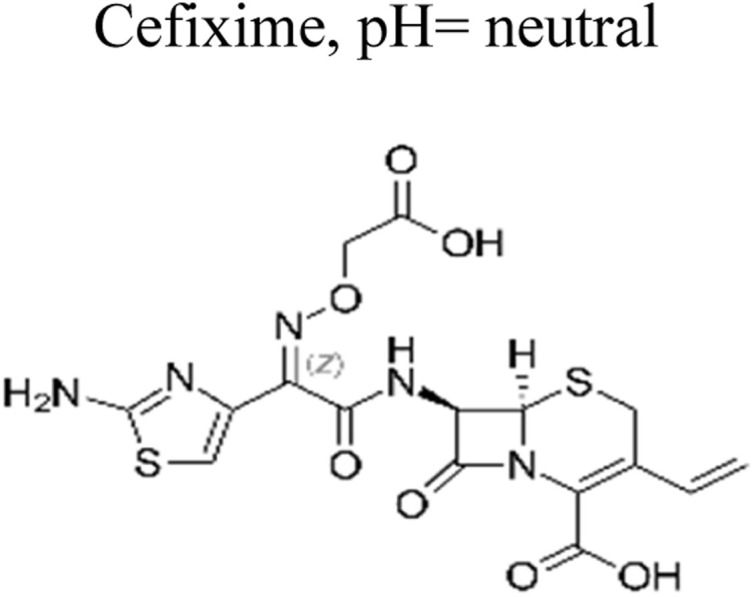	15.22 mg L^−1^	Sunlight	90%	3 h	172
7	Ag/ZnNb_2_O_6_@SC_3_N_4_	40 mg	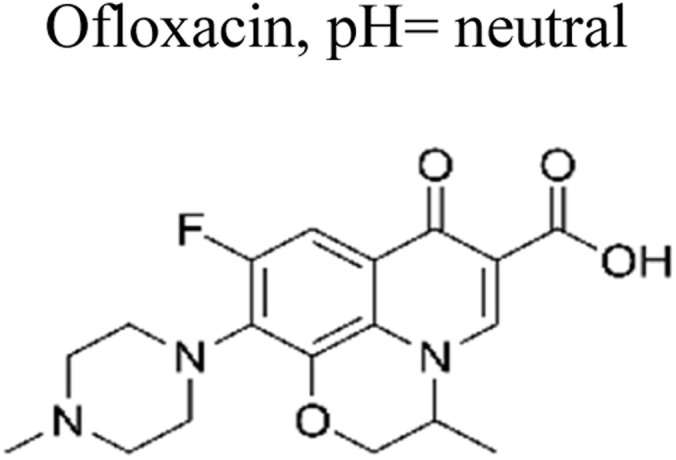	10 mg L^−1^	Tungsten lamp (300 W)	98%	1 h	171
8	Bi_3_O_4_Cl/BiOCl	1000 mg	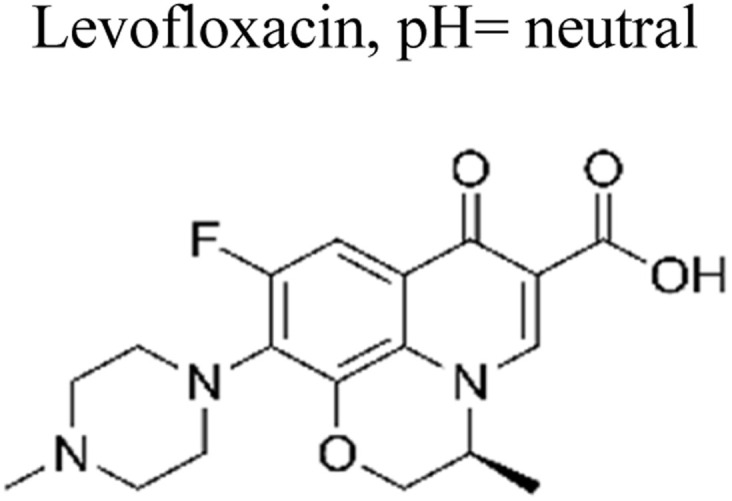	10 mg L^−1^	Compact fluorescent lamps (85 W)	87%	3 h	173
9	TiO_2_/GO/Chitosan	327 mg	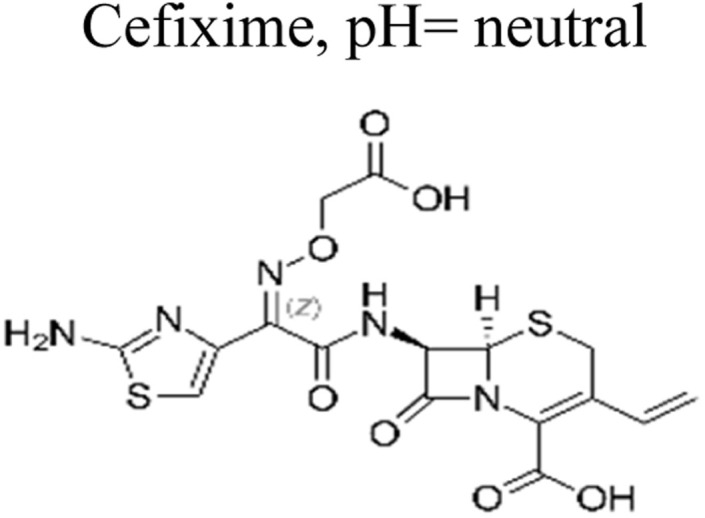	20.27 mg L^−1^	Halogen lamp (300 W)	95%	1 h	174
10	g-C_3_N_4_/Bi_3_TaO_7_	500 mg	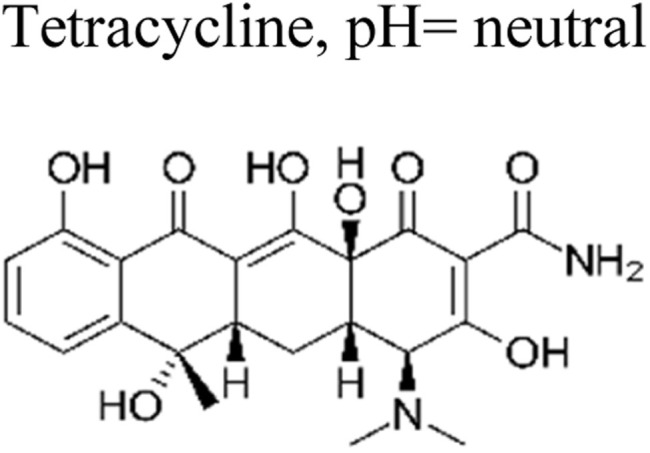	10 mg L^−1^	Xenon lamp (300 W)	89.2%	1.5 h	175
11	Ag/AgIn_5_S_8_	300 mg	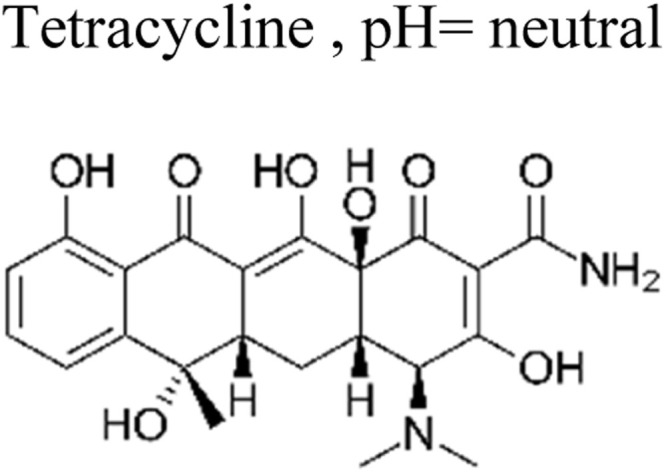	10 mg L^−1^	Xenon lamp (300W)	95.3%	2 h	176
12	Bi_2_WO_6_/Ta_3_N_5_	40 mg	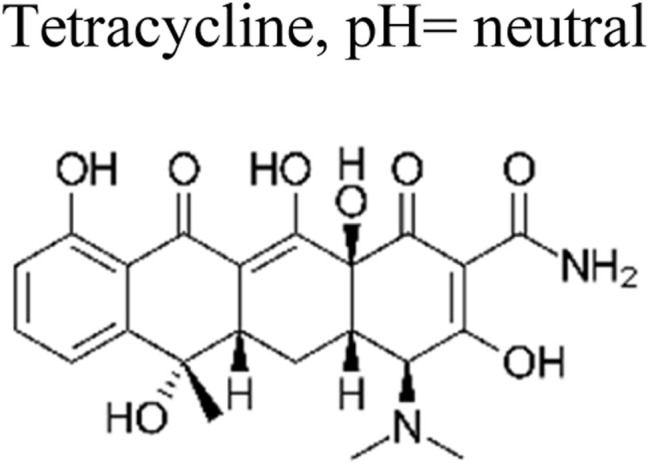	20 mg L^−1^	Xenon lamp (300 W)	86.1%	2 h	177
13	CoBi_2_O_4_	800 mg	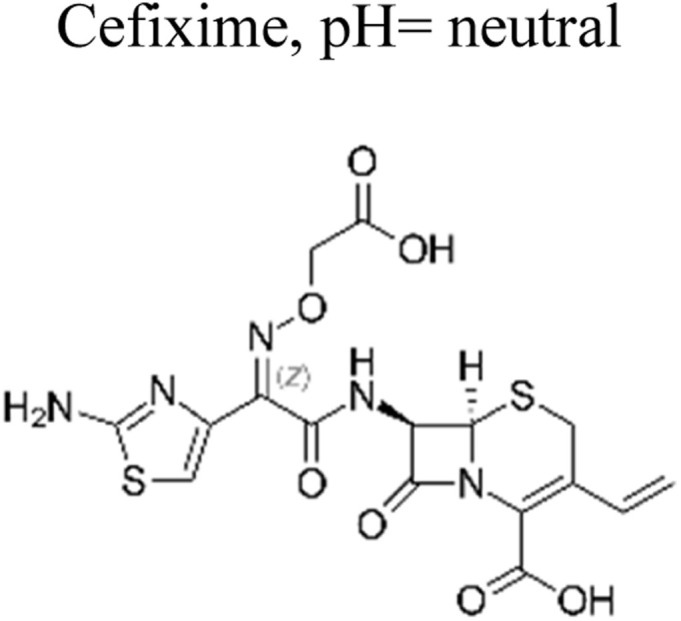	5 mg L^−1^	Tungsten lamp (200 W)	86%	6 h	178
14	PtNPs@SiO_2_@TiO_2_	100 mg	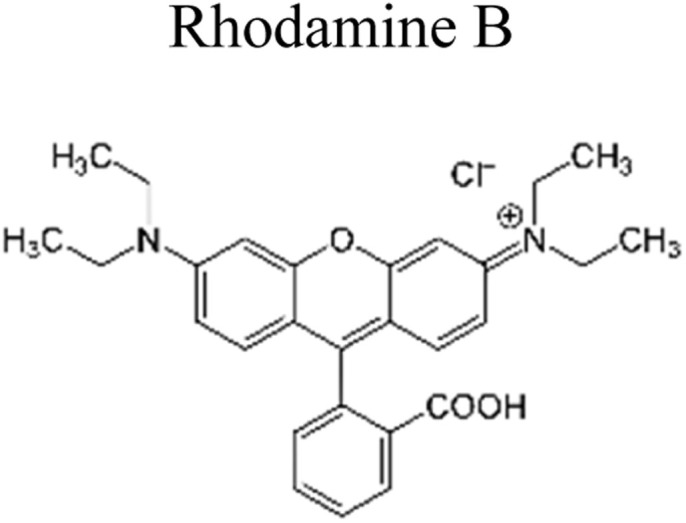	7 mg L^−1^	Xenon lamp (300 W)	61%	2 h	179
15	Ag/TiO_2_	1000 mg	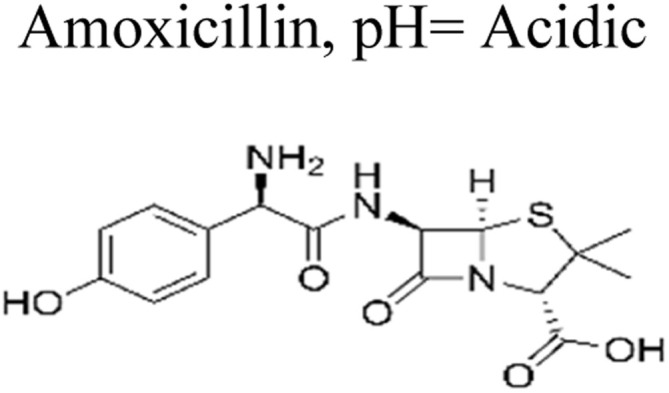	20 mg L^−1^	Tungsten–halogen lamp (500 W)	63%	5 h	180
16	r-GO/Au NPs/m-TiO_2_	5 mg	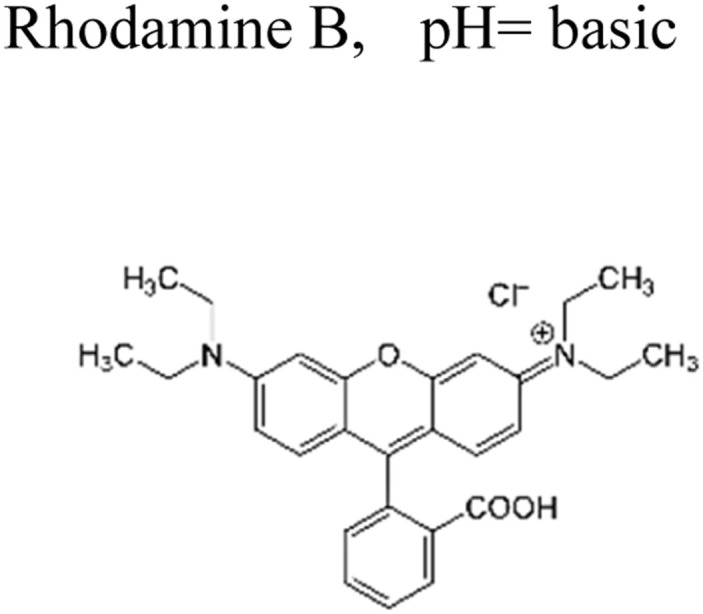	0.7 mg L^−1^	Mercury lamp (300 W)	97%	5 h	181
17	Au–Ag NPs@TiO_2_@Fe_3_O_4_ NPs	80 mg	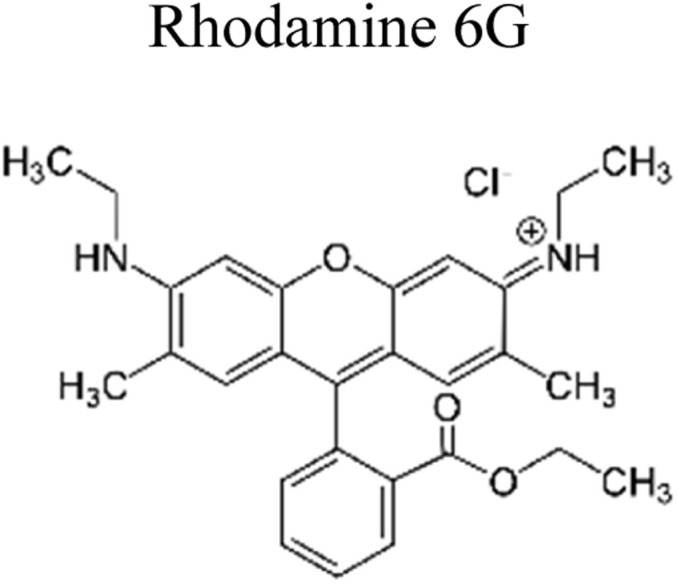	30 mg L^−1^	Xenon lamp (150 W) high pressure	100%	1 h	182
18	Nb_2_O_5_/g-C_3_N_4_	10 mg	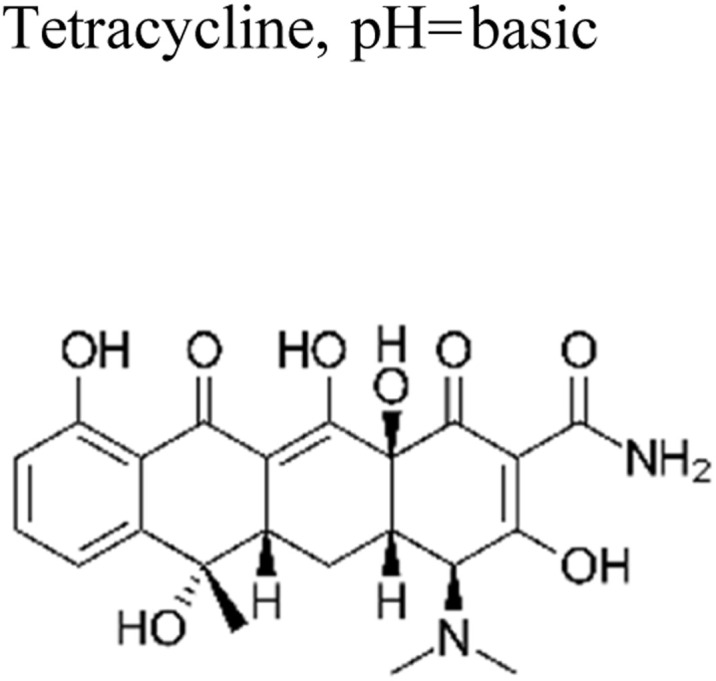	10 mg L^−1^	Xenon lamp (300 W)	76.2%	2.5 h	183
19	Au/TiO_2_ nanosheet	—	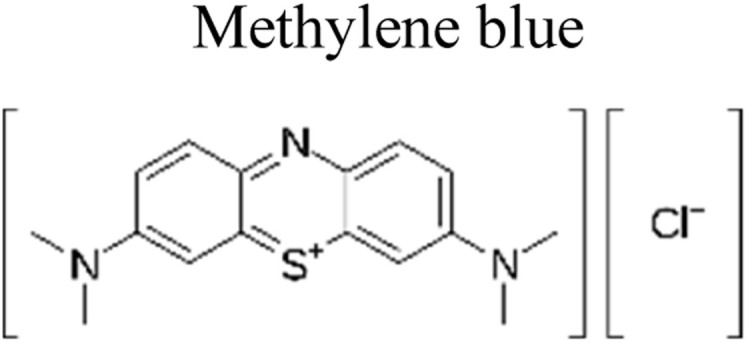	10 mg L^−1^	Xenon lamp (300 W)	66%	2 h	184
20	Ag–TiO_2_	100 mg	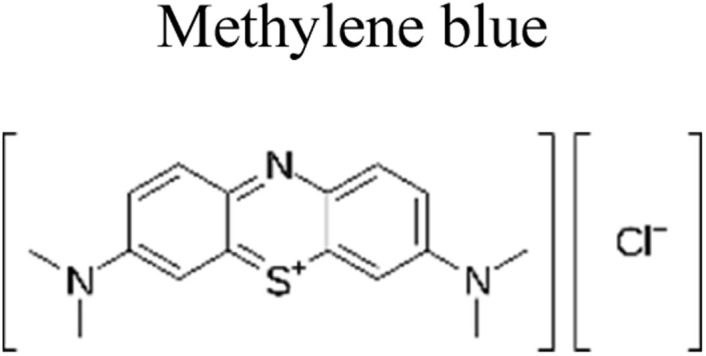	10 mg L^−1^	Xenon lamp (300 W)	50%	2.5 h	185
21	g-C_3_N_4_/TiOF_2_	300 mg	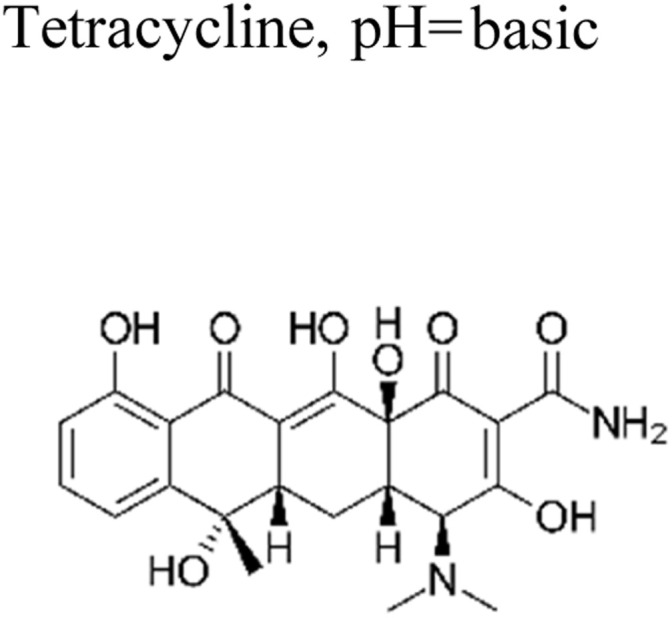	10 mg L^−1^	Xenon lamp (500 W)	85.5%	2.5 h	186
22	Bi_2_S_3_/doped g-C_3_N_4_	200 mg	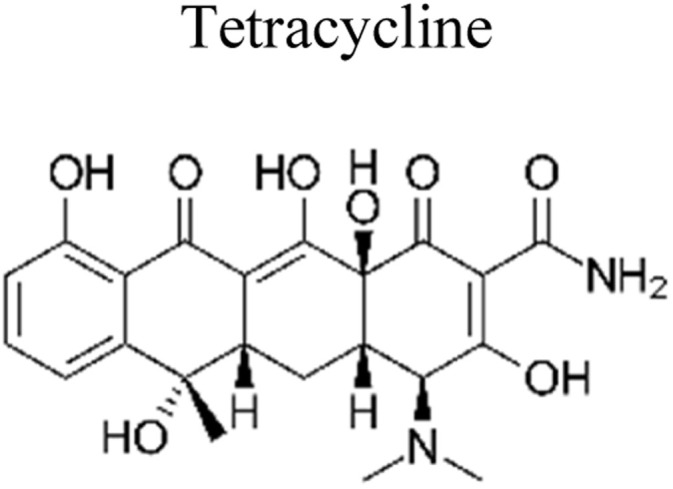	50 mg L^−1^	Halogen lamp (80 W)	99.9%	0.33 h	187

## 6. Pesticides

**Fig. 9:**
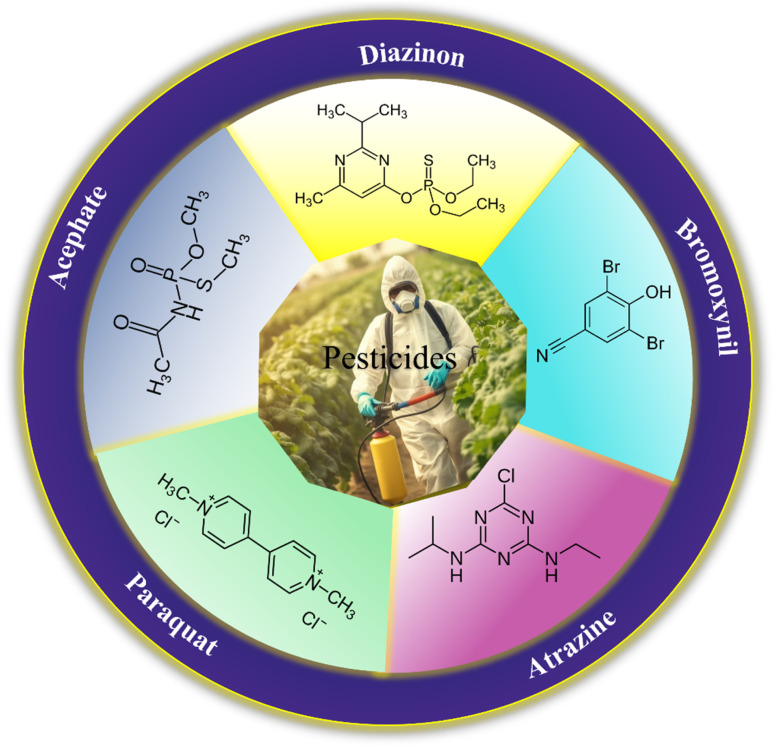
Various pesticides which are commonly utilized in agricultural applications.

**Fig. 10:**
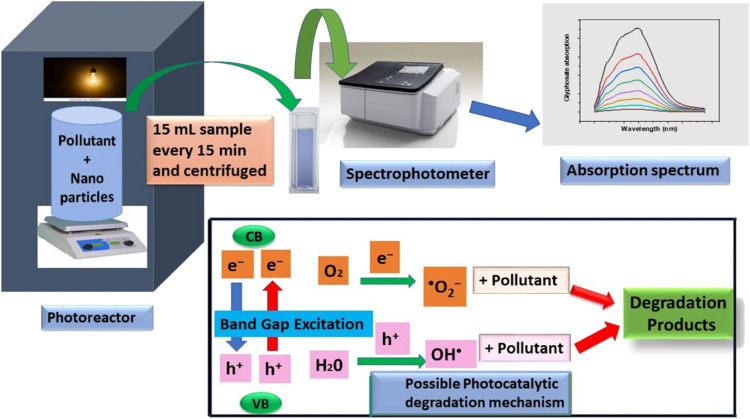
General mechanism for photocatalytic degradation of pesticides. Reproduced with permission from ref. 95, Copyright 2023, MDPI.

**Fig. 11:**
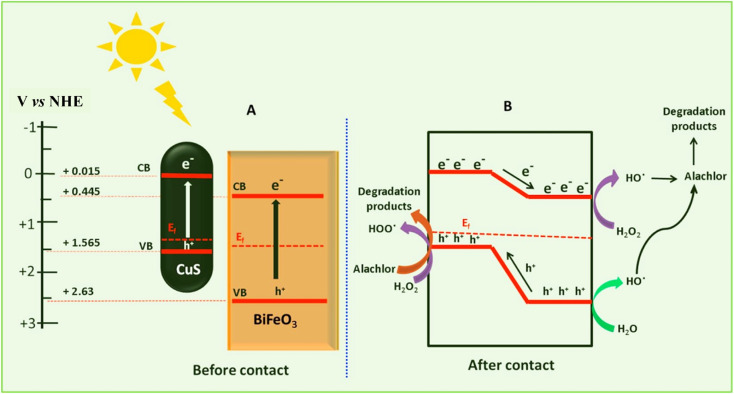
Proposed mechanism for photocatalytic degradation of alachlor. (A) Before contact and (B) after the contact. Reproduced with permission from ref. 96, Copyright 2018, Elsevier.

Pesticides are considered necessary in contemporary agriculture and pest management since they surely play a crucial part in maintaining the world's nutrition.^89^ Nevertheless, the widespread and continuous use of pesticides has generated significant apprehension over the ecological consequences and the possible risks they provide to both the environment and human well-being. In the perspective of the global endeavor to reconcile agricultural production with environmental sustainability, there is a pressing need for inventive solutions to address the detrimental consequences arising from the presence of residues from pesticides (Fig. 9). In recent years, the use of photocatalytic degradation has emerged as a feasible and sustainable technological approach for the cleanup of ecosystems polluted with pesticides.^90,91^ This conceptual technique exploits the inherent characteristics of some semiconductor photocatalysts, such as TiO_2_, ZnO, CdS, and CuS, to utilize energy from sunlight or artificial light sources.^92,93^ This process triggers a series of redox reactions that efficiently convert pesticide molecules into non-toxic substances. The use of photocatalytic degradation in addressing pesticide contamination is a novel and versatile approach^94^ which illustrates a typical schematic diagram of photocatalytic degradation of pesticides^95^ (Fig. 10). This method provides a very effective approach for the elimination of pesticide residues from several environmental mediums, including water, soil, and air, thereby mitigating the potential harm to environmental and human health. Also, the photocatalytic process is in accordance with the principles of sustainability since it employs renewable energy sources that are environmentally friendly to facilitate the degradation processes. In this study, Bhoi *et al.* reported a significant degradation rate of 95% for alachlor during a span of 60 min. This degradation was achieved by the use of a Type-II CuS/BiFeO_3_ heterojunction, which was subjected to visible light irradiation (Fig. 11). Within this heterojunction, the migration of holes from the valence band (VB) of BiFeO_3_ (BFO) to the valence band of CuS occurs, while the transfer of electrons from the conduction band (CB) of CuS to the conduction band of BFO takes place. The spaces inside the valence band of copper sulfide (CuS) may subsequently facilitate the direct oxidation of alachlor. Furthermore, hydroxyl (˙OH) radicals may be generated by the cleavage of water molecules inside the valence band of copper sulfide (CuS) and by the activation of hydrogen peroxide (H_2_O_2_). Scavenger examinations provide evidence that substantiates this outcome.^96^ In another investigation, it was demonstrated that when exposed to visible light (150 W Xe lamp), 95% of diuron (10 ppm) decomposed within 3 h. The Type-II heterojunction between CuS and Bi_2_W_2_O_9_ significantly enhances photocatalytic efficiency. Empirical evidence from trapping experiments supports the significant involvement of hydroxyl radicals and holes in the photoreaction, as reported.^97^ For the elimination of lindane, a BiOI_0.5_Br_0.5_ photocatalyst, synthesized using the co-precipitation technique, was employed. The photocatalytic activity of BiOI/BiOBr proved highly effective in degrading lindane under visible light exposure. Specifically, at a pH of 8, nearly 98% of lindane was successfully eliminated. The optimization of the photocatalyst dosage and pH were conducted in this work, determining the optimal conditions as 1 g L^−1^ and a pH of 8. Several semiconductor heterojunctions have been identified for the purpose of photocatalytic pesticide destruction.^98^Table 2 summarizes several semiconductor heterojunctions used in the photocatalytic degradation of pesticides.

**Table 2:** Photocatalytic degradation of various pesticides

S. no.	Photocatalyst	Catalyst mass (g L^−1^)	Pesticides	Pollutant mass (mg L^−1^)	Light source	Time (min)	Removal	Ref.
1	S–TiO_2_@rGO	0.8	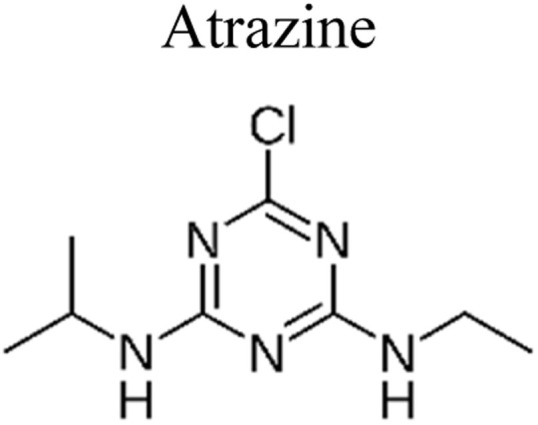	20	300 W Xe lamp	20	100%	188
2	S–TiO_2_	1	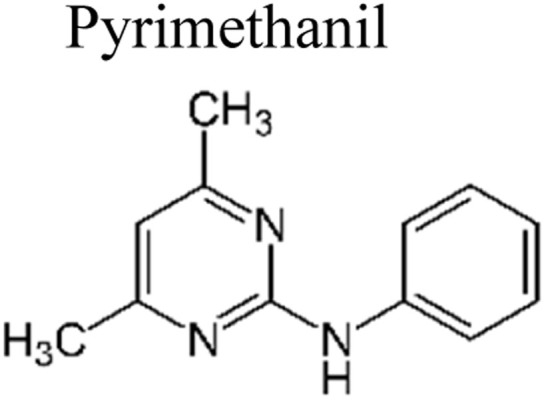	1	LED lamp	25	100%	189
3	CuO/TiO_2_/PANI	0.45	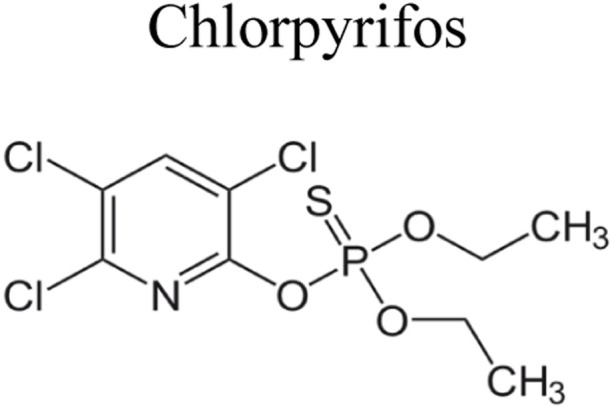	5	Visible light	90	95%	190
4	CuS/Bi_4_Ti_3_O_12_	0.25	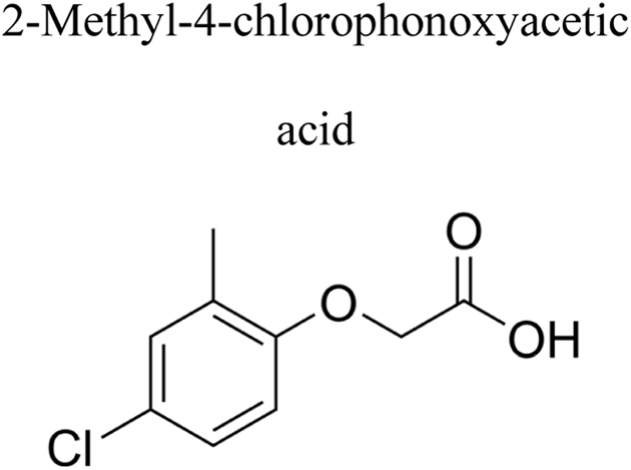	10	Visible light	180	95%	191
5	Fe_3_O_4_@SiO_2_@mTiO_2_	0.1	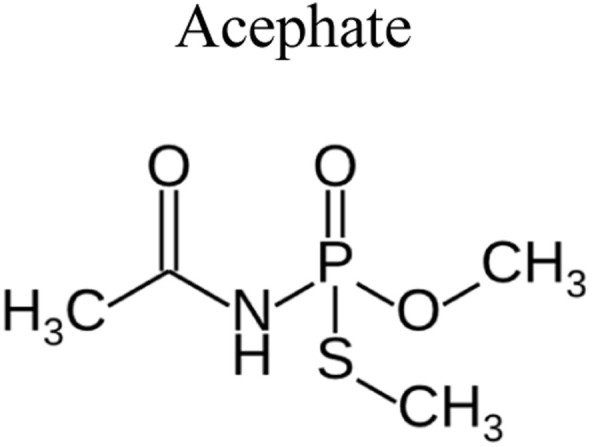	100	UV-visible	80	100%	192
6	Cs–TiO_2_	1	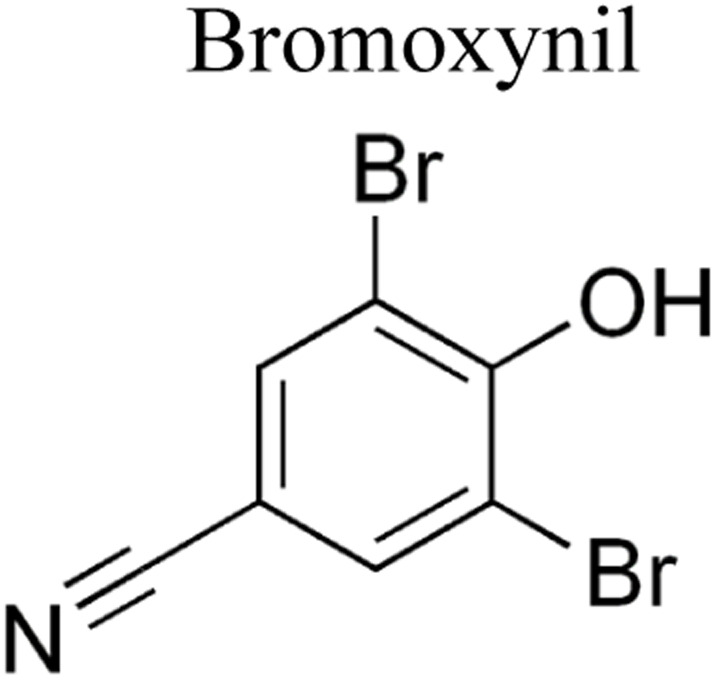	10	LED	120	100%	193
7	Cu–TiO_2_/SBA-15	0.5	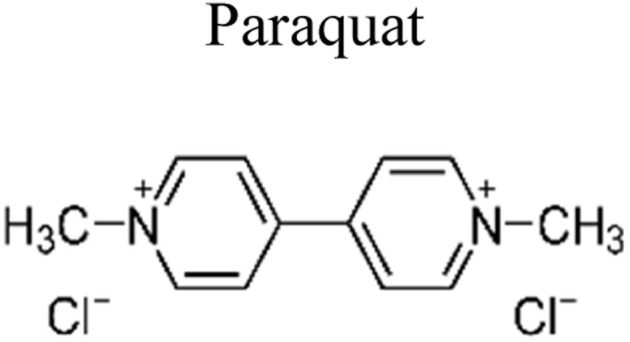	40	Visible light	240	55%	194
8	CuS@rGO	0.8	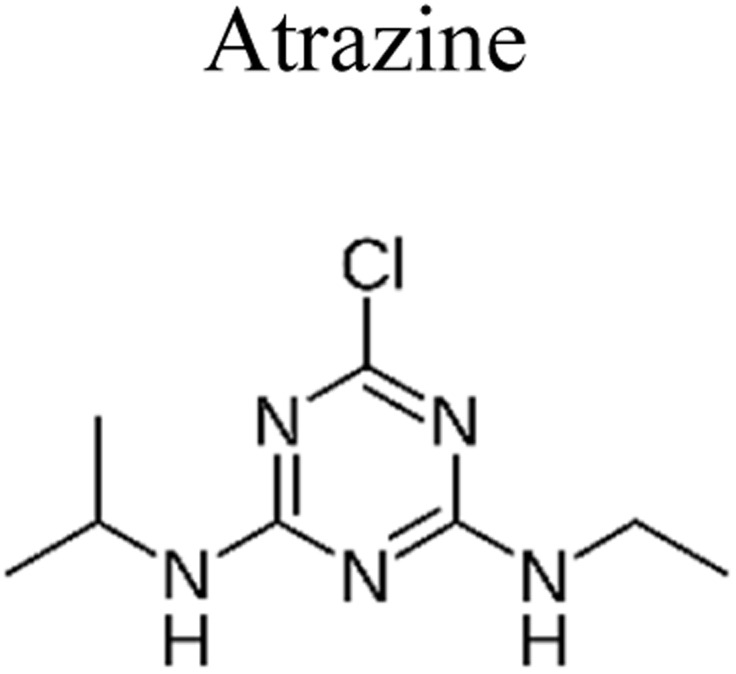	50	Visible light	20	100%	195
9	CuS/Bi_2_WO_6_	0.4	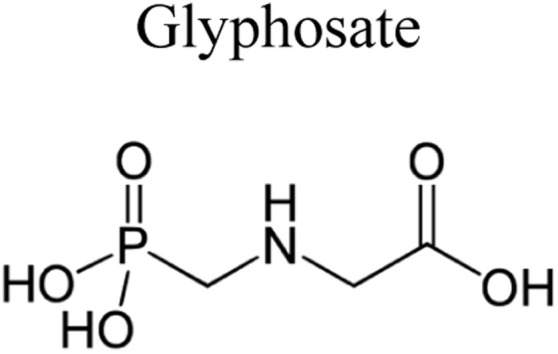	—	Visible light	180	85.9%	196
10	TiO_2_ nanowire	1	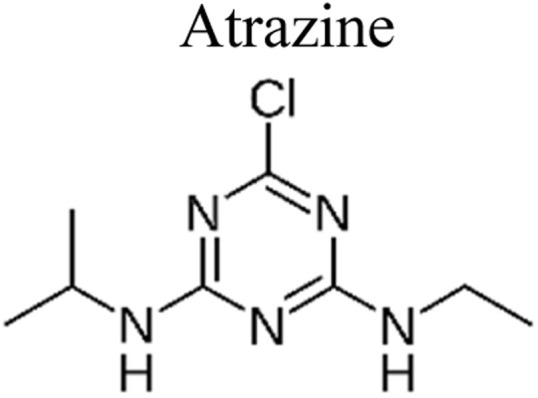	5	UV-light	60	57%	197
11	TiO_2_/carbon composite	0.5	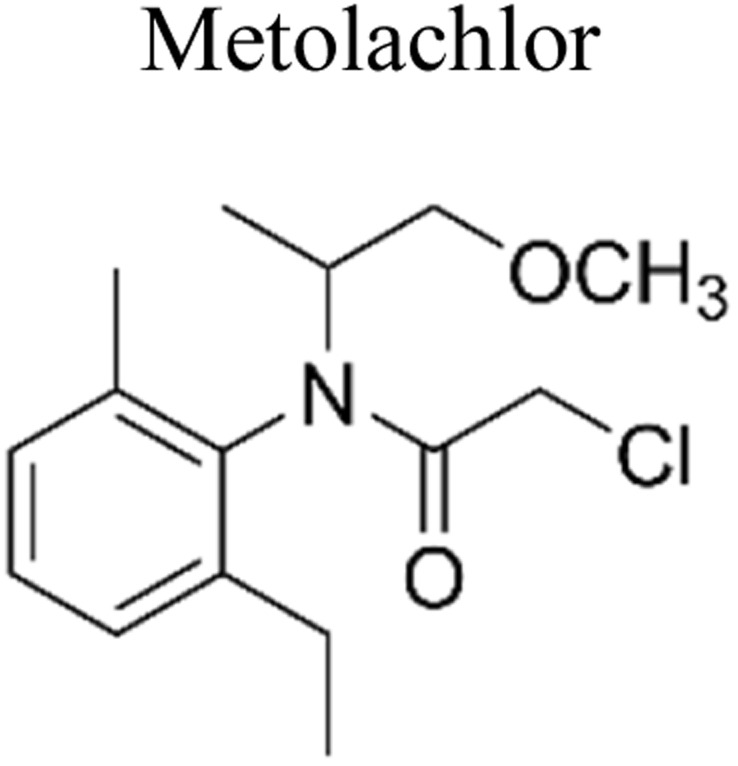	20	UV light	30	100%	198
12	Fe_3_O_4_@SiO_2_@mTiO_2_	0.1	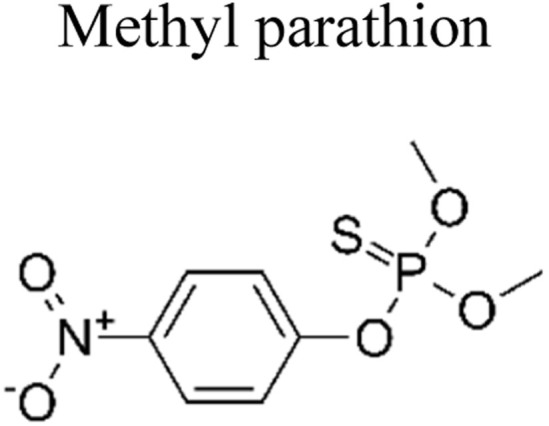	100	UV light	45	100%	192
13	TiO_2_-silica gel	56	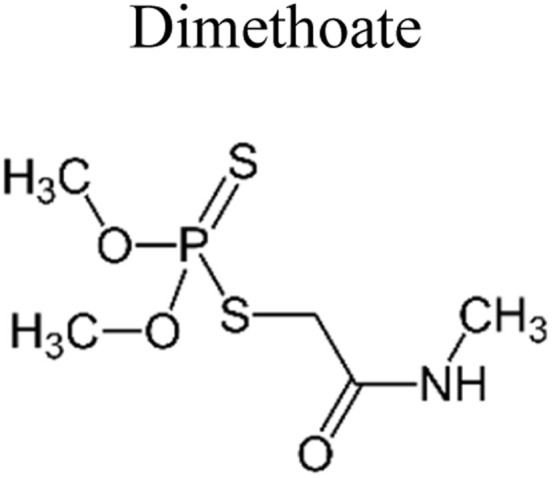	22.9	UV light	60	100%	199
14	Fe_3_O_4_/CdS–ZnS	0.0033	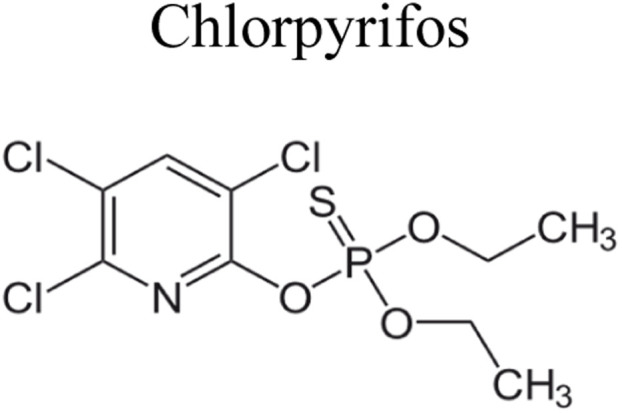	10	Visible light	60	94.5	200
15	ZnO@CdS	0.001	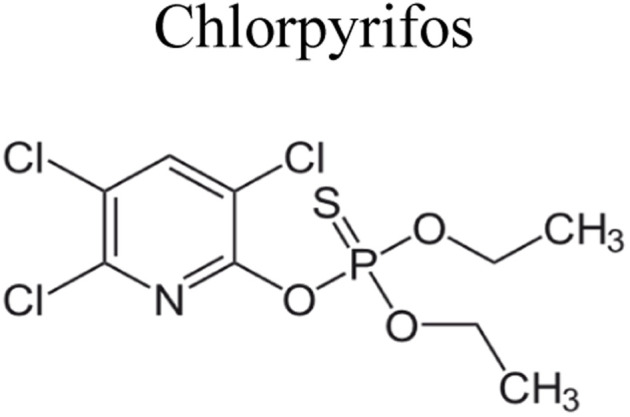	2	Visible light	360	91	201

## 7. Green fuel production

The extensive exploitation and widespread use of fossil fuels have led to increasingly severe environmental pollution and energy shortages. Solar energy, which is abundant and renewable on our planet, offers a viable solution to the ongoing energy crisis.^99–101^ The field of photocatalytic technology is experiencing a significant surge in research efforts aimed at harnessing sunlight to produce storable chemical energy, with a particular focus on the clean and highly efficient production of hydrogen energy. Hydrogen energy is regarded as an environmentally sustainable energy source due to its unique byproduct: water, without any associated emissions of carbon dioxide or other harmful pollutants. Hydrogen (H_2_) boasts a calorific value of 141 kJ g^−1^, surpassing the calorific values of traditional fossil fuels such as oil (46 kJ g^−1^) and natural gas (55 kJ g^−1^). These characteristic positions hydrogen as a promising clean and sustainable energy alternative.^102,103^ However, it's crucial to acknowledge that a significant majority, over 90%, of conventional hydrogen production heavily relies on fossil fuels, exacerbating the aforementioned environmental concerns. Therefore, expediting the development of sustainable technology for green hydrogen production is imperative.^104,105^

The process of water photolysis, which involves the splitting of water molecules into hydrogen (H_2_) and oxygen (O_2_), is widely recognized as a highly promising approach for addressing the increasing global energy requirements and environmental concerns.^106–109^ However, Bie *et al.*^110^ conducted a comprehensive analysis of various factors affecting photocatalytic total water splitting technology, both from theoretical and practical perspectives. They concluded that this technology poses significant challenges, both presently and in the future. On the other hand, simultaneous degradation of pollutants and generation of hydrogen through photocatalysis is regarded as a promising and attainable area of focus in the advancement and use of dual-function photocatalysts.^111^

### 7.1. Photocatalytic mechanism of hydrogen generation and degradation

**Fig. 12:**
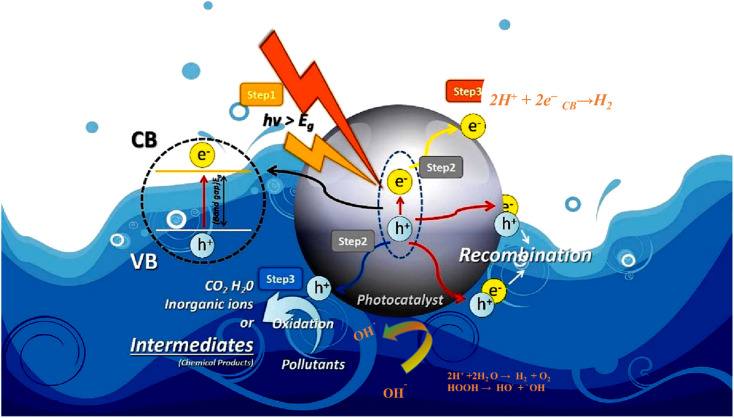
Schematic diagram for photocatalytic mechanism of hydrogen generation and pollutant degradation. Reproduced with permission from ref. 112. Copyright 2023, Elsevier.

The photocatalytic processes of both photocatalytic hydrogen generation *via* water and photocatalytic degradation of pollutants are distinct phenomena. However, when these two processes are coupled, as in the case of synergistic pollutant degradation, the overall mechanism comprises the following sequential steps: The three main processes involved in the photocatalytic activity of semiconductor materials is shown^112^ in Fig. 12. (i) The absorption of light, (ii) the generation and migration of electron–hole pairs, and (iii) the occurrence of surface redox reactions.^11,113^ Semiconductor materials possess a significantly narrower band gap between the valence band (VB) and the conduction band (CB) when compared to insulators. This narrower band gap enables photons with energy equal to or greater than the semiconductor's band gap energy to excite and mobilize electrons within the material, setting the stage for subsequent photocatalytic reactions. Notably, visible light, which constitutes a substantial portion of sunlight (46%), surpasses ultraviolet light (5%) and is more readily absorbed and utilized in contrast to infrared light (49%). Consequently, enhancing the light absorption capabilities of photocatalytic materials for visible and even near-infrared light becomes paramount.^114–117^ Once electron–hole pairs are generated, it becomes essential for these photogenerated carriers to have a sufficiently extended lifetime, allowing them to migrate to the catalyst surface or reaction sites and participate in reactions. During this migration process, photogenerated electrons and holes tend to recombine with each other, and the rate of this recombination plays a pivotal role in determining photocatalytic performance. It's worth noting that materials with strong light absorption properties may not always exhibit excellent photocatalytic performance. Surface redox reactions occur subsequent to the migration of photogenerated carriers to the reaction sites. At this stage, protons in water get converted to hydrogen gas by photogenerated electrons, while pollutants are sequentially broken down into other intermediates or transformed into carbon dioxide and water by photogenerated holes or other potent oxidative species. The specifics of the decomposition mechanism and the properties of pollutants, alongside the presence of co-existing substances, emerge as key factors influencing on this process.^103,110,118^

### 7.2. Challenges and difficulties of dual-functional photocatalyst

The significance of conducting multiple investigations into photocatalytic hydrogen synthesis from water and the degradation of pollutants through photocatalysis is evident. However, our current understanding of synergistic catalysis remains insufficient. The main challenge in achieving dual-functional photocatalysis is that traditional photocatalytic hydrogen production typically takes place in an anaerobic environment, where oxygen interferes with the capture of photogenerated electrons necessary for proton reduction, thereby affecting the subsequent hydrogen production reaction. On the other hand, photocatalytic pollutant degradation usually occurs in aerobic conditions, where oxygen often acts as an electron trap, facilitating the separation of electron–hole pairs and transforming them into superoxide radicals that can further accelerate pollutant degradation and sustain the reaction.^119–121^ Combining these two processes necessitates not only an anaerobic environment, appropriate reaction conditions, and containers but also the assurance that each side reaction will not interfere with the other. Furthermore, it is essential to maintain the efficiency of both hydrogen generation and pollutant degradation throughout the process. Addressing this complex issue is of paramount importance.

The dual-functional photocatalysis presents unique challenges that go beyond those faced by traditional monofunctional photocatalysis, and these challenges become even more complex under demanding conditions. In the initial phase of light absorption, materials with narrow band gaps exhibit strong light absorption capabilities. However, their limited utilization of light energy and inherent instability can negatively impact photocatalytic efficiency. Conversely, materials with wider band gaps are more stable but have poor absorption of visible light. For instance, metal oxides are often considered ideal for photocatalytic reactions due to their stability and antioxidative properties. Nonetheless, their wide band gaps result in the absorption of only a small portion of the solar spectrum, severely limiting solar energy conversion efficiency.^122,123^ Recent studies have demonstrated that introducing oxygen vacancies can lead to the creation of transition state energy levels, thereby narrowing the band gap and enhancing light absorption capacity. However, these oxygen vacancies can also introduce lattice distortions and alter spatial polarity, hindering the efficient transport of photogenerated carriers and reducing photocatalytic efficiency. Thus, finding methods to simultaneously achieve high light absorption and energy utilization is a challenging endeavor in current research.^124–126^

When it comes to the separation and migration of photogenerated carriers, the primary objectives include reducing the recombination of electron–hole pairs and increasing their migration rates. These goals are intricately linked to the specific compositions and microstructures of catalysts. Moreover, to achieve efficient hydrogen production while concurrently degrading pollutants synergistically, we must address additional factors. These include the characteristics of pollutants, their interactions with catalysts, solvents, and products, redox potentials, intermediates, reaction mechanisms, and the overall purpose of the treatment process. In our discussion ahead, we will delve into these factors in detail, drawing insights from the latest research cases and exploring them from two distinct perspectives: catalysts and pollutants.^11,117,127^

### 7.3. The effect of a present photocatalyst

**Fig. 13:**
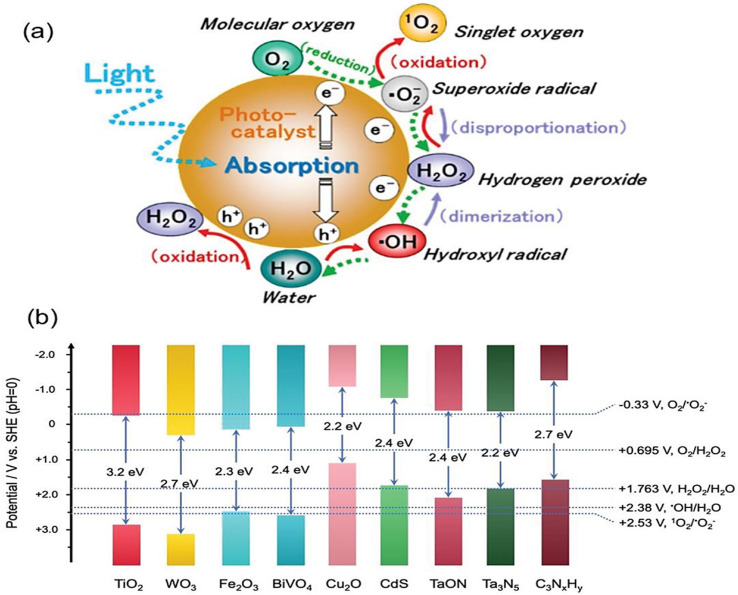
(a) ROS generated by photocatalytic redox reaction and (b) redox potential of different semiconductor photocatalyst energy level diagram. Reproduced with permission from ref. 129 Copyright 2021, Wiley.

The first step in achieving a good synergistic photocatalytic effect is to choose an appropriate and efficient photocatalytic material. Because of their strong photocatalytic capabilities, structural tunability, and reaction stability, materials such as transition metal sulphides, metal oxide, metal–organic flameworks (MOFs), and graphitic phase carbon nitride have been the focus of numerous studies.^128^ The basic requirement for the appropriate phenomenon of the photocatalytic process is the proper alignment of the redox potential and band gap. Only with a suitable band gap width, a sufficiently negative conduction band, and a sufficiently positive valence band, as well as tuneable properties, the substance that can be used for further modification studies. While the individual substances mentioned above exhibit certain advantageous and can be partially meet the redox potential and band gap requirements, as illustrated in Fig. 13. The energy level diagram,^129^ they also possess several limitations that necessitate optimization for enhanced photocatalytic performance through specific means.^130,131^

### 7.4. Metal oxide (TiO_2_)

Titanium dioxide (TiO_2_) is an extensively investigated metal oxide catalyst due to its chemical and thermal durability, non-toxicity, high reactivity, abundance, and cost-effectiveness. It nevertheless comes with certain restrictions. Because of its quick charge carrier recombination rate and a comparatively large band gap (3.2 eV), it can only absorb UV light, resulting in a poor photogenerated charge consumption rate.^132–134^ TiO_2_ stands out, however, since no other abundant, low-cost metal oxide has the same mix of qualities. It possesses conduction and valence band boundaries that span water and many water-based organic contaminants redox potentials. TiO_2_ is available in three polycrystalline phases: brookite, anatase, and rutile. The low-temperature TiO_2_ anatase phase is known to contain the most active sites owing to surface hydration. The thermodynamically more stable TiO_2_ rutile phase, on the other hand, has a narrower band gap, implying higher light absorption capability.^135,136^ As a result, efficiently managing the phase ratio is the key to maximizing TiO_2_ photocatalytic characteristics. This control enables researchers to balance the benefits of the anatase phase's active sites with the rutile phase's increased light absorption capability, leading to enhanced photocatalytic capabilities.^137^

#### 7.4.1. Metal and non-metal doping

Doping TiO_2_ with non-metal and metal ions has the potential to enhance its light absorption capabilities. Metal doping facilitates charge separation and can lead to a favourable shift in the band gap. Introducing noble metal ions onto the TiO_2_ surface can boost photocatalytic activity within the visible range of the solar spectrum, improving light absorption. However, the use of noble metals like Ag and Au raises environmental concerns due to toxicity, and their synthesis process is expensive. Additionally, metal-doped photocatalysts are prone to photo-corrosion during the photocatalytic process. Therefore, in many instances, non-metal doping with elements such as C, S, F, and N is preferred.^138^ Non-metal dopants create intermediate energy levels within TiO_2_'s band gap, reducing the catalyst's electronegativity and thus enhancing its stability. Among non-metal dopants, nitrogen is particularly suitable for TiO_2_ due to its comparable atomic size, stability, low ionization energy, and its ability to introduce an energy level slightly above TiO_2_'s valence band. Furthermore, these energy levels serve to decrease the recombination rate of photogenerated charge carriers by acting as trapping centres for photogenerated electrons.^139,140^

Substituting non-metal elements with oxygen (O) atoms in TiO_2_ was initially thought to primarily impact the VB, dominated by O, 2p states. However, it's apparent that non-metal doping can also influence the CB, mainly composed of Ti 3d states. Lighter elements such as boron (B), carbon (C), or nitrogen (N) replacing O atoms result in the depopulation of the VB by one, two, or three electrons, respectively. Consequently, the newly introduced states are expected to be progressively less tightly bound due to the smaller effective nuclear charge. Conversely, when heavier fluorine (F) is doped, its 2p states reside below the bottom of the O 2p VB. As F is monovalent, the extra electron from the O atom must transfer to vacant Ti states, self-trapping in a 3d_*xy*_ state at the Ti site, below the CB. Interstitial doping of B, C, and N into the TiO_2_ lattice allows them to bind to lattice atoms and transfer one or more electrons based on their redox potentials. This process modifies the band gap structure, as seen in both substitutional and interstitially C-doped anatase TiO_2_.^141^

Yuzawa *et al.* tried several types of reaction experiments to investigate the reaction mechanism of Pt-loaded TiO_2_ photocatalysts in the degradation of ammonia into nitrogen and hydrogen. It was revealed that the reaction activity is mostly determined by the crystalline phase, particle size, and the number of active sites on the TiO_2_ surface. These results suggest that TiO_2_ serves as a provider of active sites for the reaction, while Pt contributes a facilitating role in this process.^142^ Hence, the control of TiO_2_ particle size, pore structure, and surface properties are of utmost importance in determining its level of activity. In their study, Kumaresan *et al.* employed the sol–gel technique to fabricate mesoporous TiO_2_ materials. They effectively allocated the size of the synthesized product by using several alcohols, as well as changing the surface properties through the incorporation of different metal ions. Notably, the researchers observed that ethanol had a more influence on particle size control compared to isopropanol and 1-butanol. Furthermore, they discovered that mesoporous TiO_2_ doped with 1 wt% Ce^3+^ exhibited superior activity in compared to both the pure form of TiO_2_ and mesoporous TiO_2_ doped with other metal ions such as Zr^4+^ and La^3+^.^143^ Musa *et al.* conducted the preparation of TiO_2_ with various phases, namely anatase, rutile, and a combination of anatase and rutile phases. This was achieved through the calcination process of MIL-125-NH_2_ at various temperatures. The investigators observed significant differences in the band gap values and substantial variations in the photocatalytic activity of the photocatalysts. Specifically, they compared the efficiency of hydrogen production and pollutant degradation between two sets of photocatalysts: TA (TiO_2_ anatase) *versus* TR (TiO_2_ rutile), and NSTA *versus* NSTR (N, S-doped TiO_2_ anatase and rutile). These differences were attributed to the distinct phases of TiO_2_ used for modification, as well as the introduction of nitrogen (N) and sulfur (S) doping.^144^

### 7.5. Graphitic carbon nitride (g-C_3_N_4_)

The photocatalytic performance of unmodified g-C_3_N_4_ is greatly hindered by many aspects, such as its limited ability to absorb light, and quick recombination of electron–hole pairs generated in the photocatalytic process.^145,146^ Nevertheless, it is indisputable that g-C_3_N_4_ possesses a two-dimensional π-conjugated structure, remarkable chemical stability, and a band gap width of 2.7 eV. These features enable it to absorb light up to 460 nm, with energy levels of approximately +1.58 eV and −1.12 eV at the *E*_VC_ and *E*_CB_ positions, respectively. Consequently, g-C_3_N_4_ emerges as an appealing choice.^147,148^ Moreover, the material is cost-effective, and facile conversion into low-dimensional nanomaterials consistently establishes it as a compelling option for the development of enhanced photocatalytic material. The conduction band potential of g-C_3_N_4_ has a significantly negative value of −1.13 eV relative to the normal hydrogen electrode (NHE). This characteristic renders g-C_3_N_4_ very suitable for the generation of hydrogen. Nevertheless, the valence band potential of the material is not sufficient to directly produce hydroxyl radicals (˙OH) through water oxidation and completely oxidize organic substances, since it is only 1.57 eV compared to the standard hydrogen electrode (NHE). Consequently, extensive research has focused on adjusting the VB potential to a more positive value, albeit this endeavour presents challenges, such as decreased light absorption due to a wider band gap. As a way to enhance the oxidation features of g-C_3_N_4_, Zeng *et al.*^131,149^ accomplished an investigation that modified the valence band (VB) position of pristine g-C_3_N_4_ by employing O-doping.^150^ This modification resulted in a rise in the VB position from +1.68 eV to +1.85 eV. This shift indicates the possibility for stronger oxidation processes and an increase in the availability of electrons for reduction reactions. The study conducted by Nie *et al.*^131^ was to optimize the efficiency of photogenerated electron–hole separation in g-C_3_N_4_ and improve the photocatalytic nature of hydrogen generation and degradation of various micropollutants. The authors incorporated graphene quantum dots (GQDs) together with Mn and N co-doped TiO_2_. It is important to emphasize that graphene quantum dots (GQDs) have the ability to capture light with longer wavelengths (600–800 nm) and then emit light with shorter wavelengths (*λ* < 500 nm). This emitted light is then absorbed by manganese–nitrogen-doped titanium dioxide (Mn–N–TiO_2_) and graphitic carbon nitride (g-C_3_N_4_), resulting in an enhancement of the whole process and the total performance of light absorption.

### 7.6. CeO_2_

CeO_2_, a rare-earth oxide distinguished by its unique 4f electrons, plays a pivotal role in various technical disciplines, including catalysis, optics, electrochemistry, and adsorption.^151^ Its significance arises from its distinctive fluorite-type structure, oxygen-rich vacancies, and remarkable redox capabilities, with valence and conduction bands positioned at approximately +2.07 eV and −0.77 eV, respectively. Nevertheless, the swift recombination of photogenerated electrons and holes, inefficient light energy utilization, and a lack of selectivity pose significant challenges in the context of individual CeO_2_ materials. Consequently, extensive research efforts have been undertaken to address and optimize these issues.^152,153^ For example, one approach to enhance CeO_2_ energy band structure for more efficient photon absorption is to modify its surface properties or create heterojunctions. In a study by Dong *et al.*,^154^ CeO_2_ nanorods (NRs) were synthesized using the hydrothermal method, and their microstructure was optimized by controlling the concentration of HO^−^ ions. Among the various samples tested, sample C3, which had a surface area of 65.26 m^2^ g^−1^, exhibited the highest Ce^3+^ ratio (22.53%), the greatest oxygen vacancy concentration (0.74), and the narrowest band gap (2.28 eV). These characteristics contributed to an improved performance in hydrogen production.^154^

## 8. An integrated approach of converting micropollutant into feasible products

Due to the intrinsic features of micropollutants and the complexities of the reaction pathways, not every micropollutant, including certain inorganic ions, can be efficiently eliminated to carbon dioxide and water. The difficulty arises in the thermodynamic component, where the redox potential often falls short of the necessary conditions. Furthermore, some by-products pose environmental risks and should be avoided. In response to these challenges, some research groups choose to utilize certain pollutants as raw materials for the synthesis of important organic compounds, such as benzyl alcohol, benzylamine, and benzenethiol. Alternatively, they could investigate herbicides for non-hazardous treatment, with the goal of achieving simultaneous energy production and material generation *via* photocatalytic hydrogen production, including a synergistic conversion of organic contaminants.^155–158^

In their study, Chiu *et al.*^159^ employed benzenethiol as a sacrificial agent for the purpose of photocatalytic hydrogen production. They observed that the most favourable conversion rate of 75.9% was achieved in the UiO-66 group. This outcome can potentially be attributed to the spatial separation of holes and electrons within the structural framework of UiO-66. Despite the lack of selectivity observed in the GC-MS data for UiO-66, the catalyst Pt/UiO-66-pz exhibited both selectivity and synergistic photocatalytic activity in the synthesis of diphenyl disulfide. Remarkably, this catalytic performance was maintained even when various benzenethiol substitutes were employed in the experiments. However, it is important to note that the activity of the catalyst was slightly diminished due to thermodynamic constraints and the electrophilic effects of the functional groups. This implies that the formulation of a particular product may take into account catalysts exhibiting catalytic selectivity, thus avoiding the constraint of relying only on one reactant in the process. Benzyl alcohol is classified as a fundamental and uncomplicated alcohol within the realm of organic molecules. Its oxidation yields benzaldehyde, which holds significance as a precursor in the synthesis of high-quality chemical substances.^160^ The economic impact of photocatalytic oxidation of benzyl alcohol with simultaneous hydrogen generation becomes evident when compared to traditional organic synthesis techniques. Therefore, the development of a photocatalytic double-optimal system that is both recyclable and effective holds significant potential for the oxidation of benzyl alcohol (or other organic compounds) through photocatalysis, concurrently facilitating synergistic hydrogen generation.^161–163^ In their study, He *et al.*^164^ employed h^+^ as a means to transform benzyl alcohol into a product of significant value. This transformation was achieved through the implementation of a photocatalytic oxidation process combined with a synergistic hydrogen production reaction. The researchers utilized a Bi/ZnIn_2_S_4_ catalyst, resulting in hydrogen production rates of up to 3658.8 μmol g^−1^ h^−1^, as well as benzaldehyde yields of 1030.4 μmol g^−1^ h^−1^. Notably, the selectivity of this process approached 100% (Table 3).

**Table 3:** Synergistic photocatalytic degradation and hydrogen production

S. no.	Photocatalyst	Mass of catalyst	Contentment	Light source	Degradation	Hydrogen production	Reference
1	Cd_0.5_ Zn_0.5_S/CoP	0.005 g L^−1^	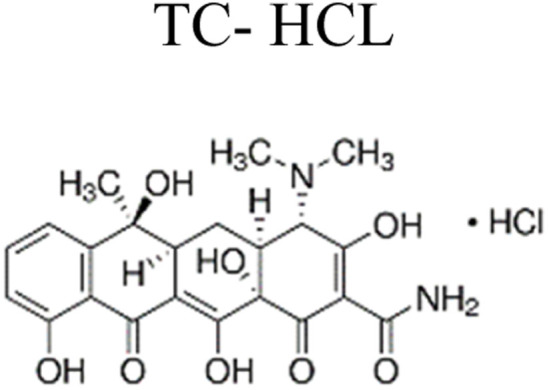	Xenon lamp (300 W)	36%	2.67 mmol g^−1^ h^−1^	202
2	Cd_0.5_Zn_0.5_S/Ti_2_C_3_	0.025 g L^−1^	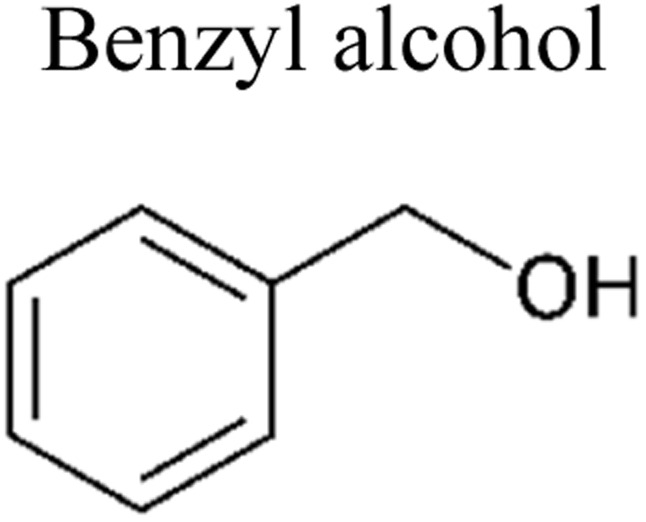	Xenon lamp (300 W)	—	5.3 mmol g^−1^ h^−1^	203
3	Au/TiO_2_	0.050 g L^−1^	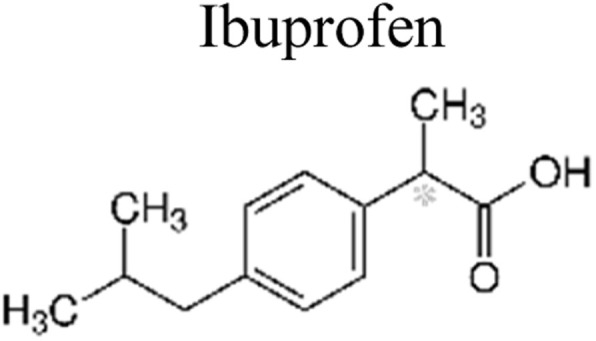	Xenon lamp (300 W)	99%	94.5 μmol g^−1^ h^−1^	204
4	Bi/ZnIn_2_S_4_	0.1 g L^−1^	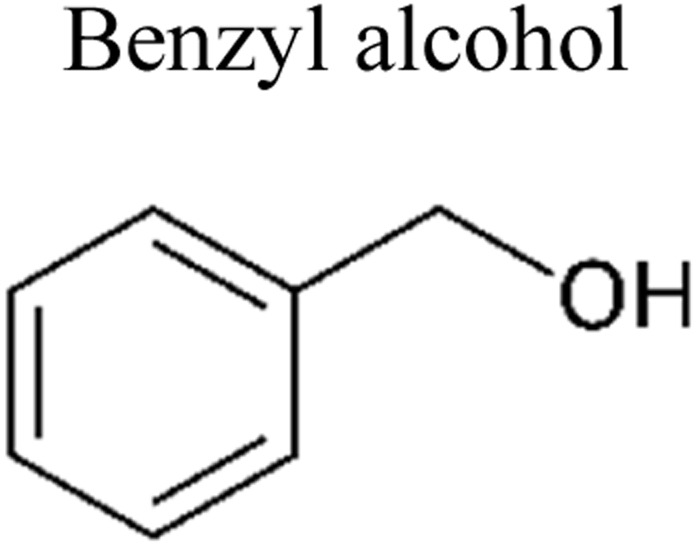	Xenon lamp (300 W)	—	3658.8 μmol g^−1^ h^−1^	164
5	Cu/UiO-66-NH_2_/ZnIn_2_S_4_	0.1 g L^−1^	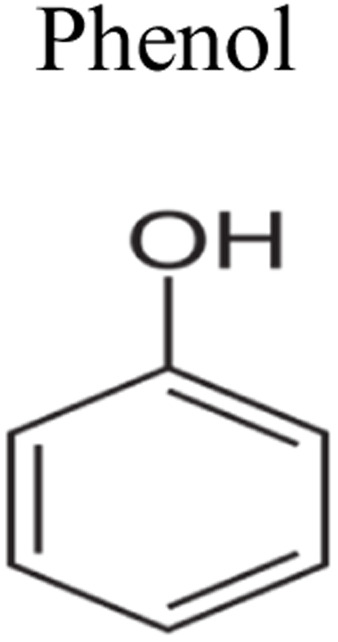	Xenon lamp (300 W)	73.1%	83.4 μmol g^−1^ h^−1^	205
6	Black P/C_3_N_4_	0.020 g L^−1^	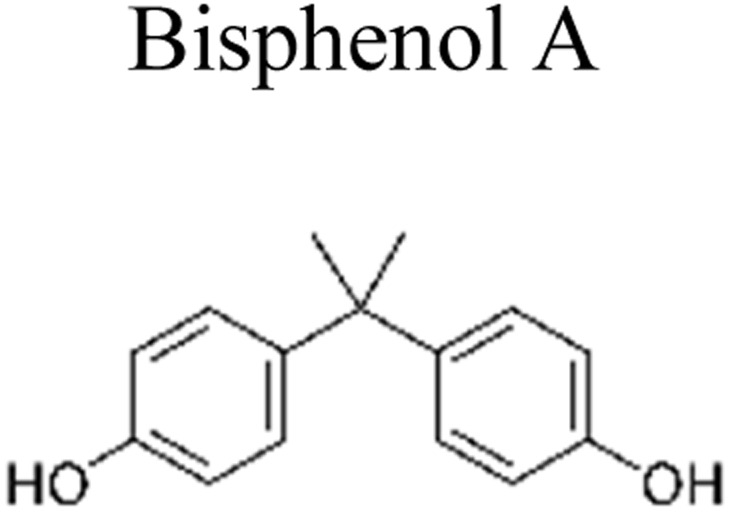	Xenon lamp (300 W)	88%	258.04 μmol g^−1^ h^−1^	206
7	g-C_3_N_4_/Pt/TiO_2_	0.010 g L^−1^	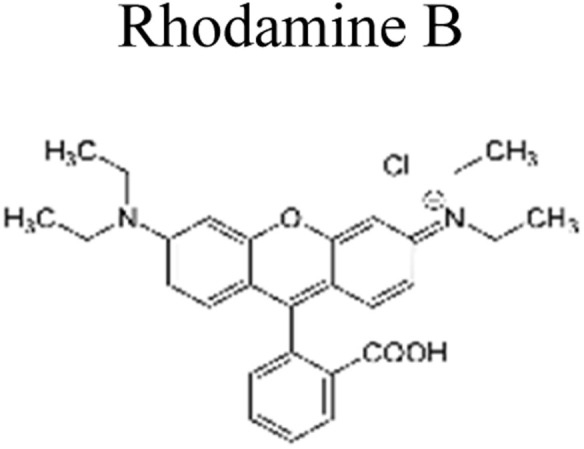	Xenon lamp (500 W)	100%	8.6 μmol g^−1^ h^−1^	207
8	CeO_2_/CeVO_4_/V_2_O_5_	0.010 g L^−1^	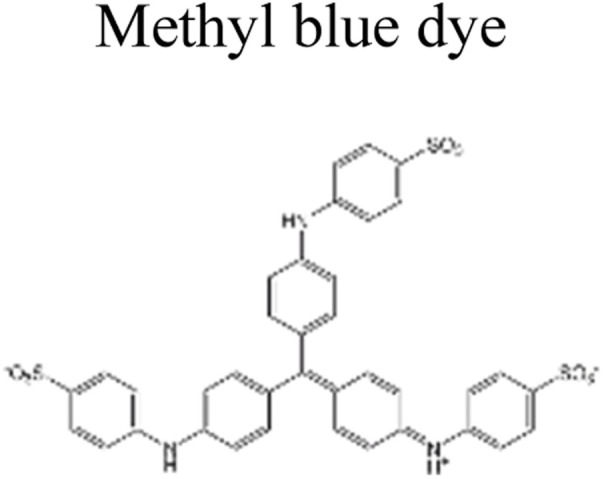	Xenon lamp (300 W)	93.53%	189.7 μmol g^−1^ h^−1^	152
9	O-doped crystalline carbon nitride	0.0125 g L^−1^	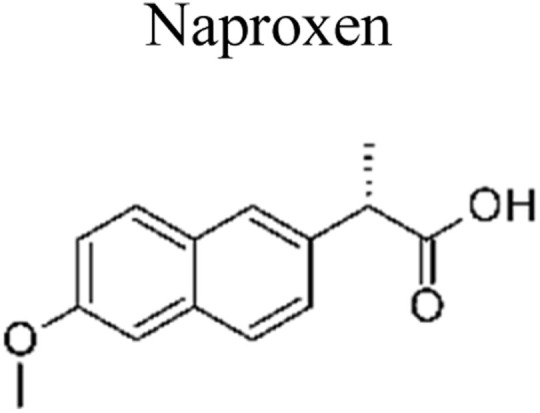	Xenon lamp (300 W)	92%	819.1 μmol g^−1^ h^−1^	208
10	Ag/g-C_3_N_4_–Ag–Ag_3_PO_4_ (110)	—	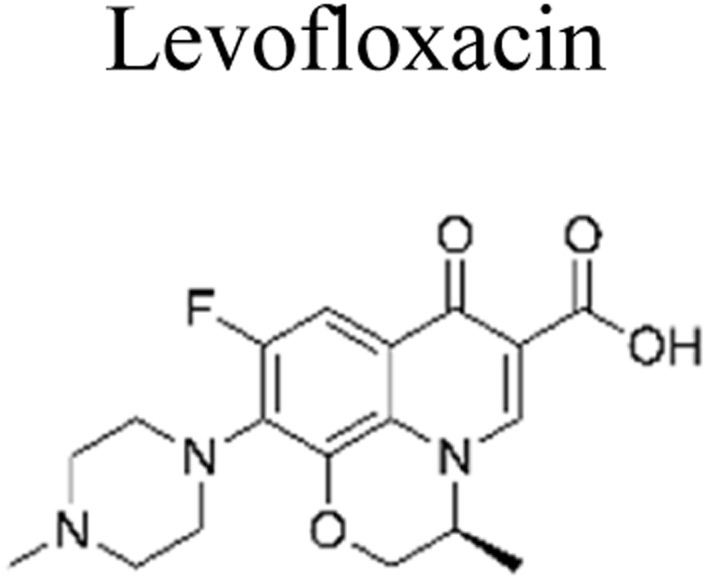	Xenon lamp (300 W)	90.44%	52.35 μmol g^−1^ h^−1^	209

Glyphosate (*N*-phosphonomethylglycine), falls under the classification of organophosphate compounds (OPC) and is extensively utilized as a non-selective postemergence micropollutant (herbicide) in global agricultural applications. Additionally, the stability, harmful nature, and migratory potential of the degradation intermediate AMPA (α-amino-3-hydroxy-5-methyl-4-isoxazolepropionic acid) surpass those of glyphosate itself. Consequently, addressing the generation of AMPA during glyphosate degradation has become a critical concern in mitigating the environmental consequences associated with glyphosate.^165^ In their study, Musa *et al.*^166^ opted to use glyphosate (*N*-phosphonomethylglycine or PMG) as the herbicide of choice for their photocatalytic tests. The investigators proceeded to analyze the relative proportions of PMG and the decomposition products, namely glycine, formic acid, and AMPA, generated in the solution. This analysis spanned across several TiO_2_ phases. Among the phases investigated, it was observed that the rutile phase exhibited limited activity in the degradation of PMG. While the performance of both pristine and doped mixed phases (consisting of both rutile and anatase) was enhanced, the resultant mixed products included formic acid, glycine, and AMPA, with PMG persisting in the system.^166^ AMPA, an intermediate of PMG, is known for its adverse environmental impact, primarily involving damage to the carbon–nitrogen bond in PMG. The N, S-doped anatase (NSTA) phase demonstrated both efficient degradation of PMG and the generation of environmentally safe by-products, namely glycine and formic acid. This efficiency was attributed to the catalytic properties of NSTA, facilitating the cleavage of the C–P bond in PMG, leading to the production of glycine. It is important to note that the cleavage of the C–N bond was somewhat hindered in this process. Additionally, the findings highlight the catalyst's selectivity towards the breakdown route of the pollutant. Furthermore, evaluating the toxicity of the pollutant intermediates and their production process is crucial. The research group conducted similar experiments on various pesticides and observed a potential correlation between the solubility of different herbicide in water and the yield of H_2_. Specifically, the herbicides PMG, exhibiting high solubility and hydrophilic properties, demonstrated significantly greater efficiency in hydrogen production.^166^ This emphasizes the need for developing dual-functional photocatalytic devices that prioritize the characteristics of the pollutant and treatment requirements as the starting point for development.

## 9. Future prospect

The future direction of photocatalytic degradation of micropollutants and its role in green fuel production from wastewater is poised to undergo significant advancements, Photocatalytic H_2_ production harnesses the potential of sunlight and water, utilising semiconductors to generate H_2_. Semiconductors have the ability to utilize both UV and visible light from the solar spectrum to generate photoexcited electrons and holes, which can then be used in H_2_ production and micropollutant degradation reactions. The investigation of the most efficient dual-functional photocatalysts which are capable of producing hydrogen (H_2_) and degrade pollutants at the same time has transcended conventional material limitations. Whereas metal oxides, sulfides, and nitrides exhibit their limited light absorption and rapid charge carrier recombination pose significant obstacles to efficiency. Researchers are using a comprehensive strategy to address these obstacles. This complex approach seeks to accomplish two key goals: first, increasing visible spectrum light absorption to maximize solar energy utilization; and second, preventing charge carrier recombination to guarantee active carrier participation in both pollutant degradation and H_2_ evolution reactions. Many different techniques are being used, such as the integration of dopants (metallic and non-metallic), precise surface changes, morphological engineering, light sensitization for increased absorption, metal ion placement strategically, and deposition of noble metals to promote charge transfer and the creation of heterojunctions to effectively separate charges. In this decade, there has been a rise of interest in investigating the morphologies of components in photocatalyst systems, which range from zero-dimensional (0D) to one-dimensional (1D) and two-dimensional (2D) forms. These heterogeneous structures with varying dimensionalities have emerged as prospective options for improving photocatalytic processes. Morphologies with porosity or decreased dimensions offer various advantages, most notably by increasing surface area, allowing for better light absorption and producing more active sites for photocatalytic processes. As a result, it has become critical to better understand the impact of morphology on both single- and multi-component photocatalytic systems.

Driven by key developments in advanced materials and a focus on futuristic dual-functional photocatalysis. The following keywords highlight the anticipated trends in this field.

### 9.1. Advanced materials revolution

#### 9.1.1. Perovskite-based and LDH photocatalysts

The integration of perovskite materials holds great promise for enhancing the efficiency, high surface area and selectivity of photocatalysts in micropollutant degradation.

#### 9.1.2. Organic–inorganic Hybrids

Innovations in designing hybrid materials combining organic and inorganic components will contribute to improved photocatalytic performance.

#### 9.1.3. Quantum dots and carbon quantum dots

The use of quantum dots as photocatalytic agents is expected to revolutionize the degradation process, offering enhanced efficiency and specificity.

### 9.2. Tailoring for specific challenges

#### 9.2.1. Customized approach

Researchers will focus on tailoring materials for the unique challenges posed by different micropollutants, particularly antibiotics and pesticides.

#### 9.2.2. Specific materials

Specialized materials will be designed to address the specific nature of emerging pollutants, ensuring a more effective and targeted degradation process.

### 9.3. Futuristic dual-functional photocatalysis

#### 9.3.1. Large-scale production

The future of syngenetic photocatalysis envisions large-scale production facilities, integrating advanced materials for efficient micropollutant degradation.

#### 9.3.2. Clean water and hydrogen production

The dual functionality of photocatalysis will not only purify water but also contribute to the sustainable production of hydrogen, aligning with the broader goals of clean energy and environmental conservation.

### 9.4. Established industry integration

#### 9.4.1. Industry adoption

The continuous improvement of photocatalytic technologies will facilitate their integration into established industries, ensuring widespread application for water treatment and green fuel production.

#### 9.4.2. Industrial-scale implementation

The transition from laboratory-scale experiments to industrial-scale production will be a key milestone, making photocatalysis a mainstream technology for addressing water pollution challenges.

### 9.5. Sustainability and clean future

#### 9.5.1. Ongoing research

The relentless pursuit of advanced materials demonstrates an intense desire to constant enhancement, therefore solidifying photocatalysis as a state-of-the-art technique.

#### 9.5.2. Environmental impact

The focus on sustainability will be integral, with the aim of mitigating the impact of micropollutants on ecosystems and contributing to a cleaner, more sustainable future.

## 10. Conclusion

This comprehensive review article delves into the dynamic field of photocatalytic degradation of micropollutants, elucidating its pivotal role in the generation of hydrogen energy from wastewater. Within this study, we've scrutinized recent advancements in the photocatalytic degradation of pharmaceutical wastewater and pesticides, alongside the exploration of dual-functional photocatalysts. Our principal objective has been to unravel the potential applications of this technology while shedding light on its progress. Photocatalytic total water splitting has, for a considerable time, posed a formidable challenge, necessitating meticulous scientific and practical considerations. Nonetheless, the pursuit of photocatalytic hydrogen production combined with simultaneous pollutant degradation offers a more feasible and enticing solution. This cutting-edge technology has added benefit of producing hydrogen in addition to effectively purifying water through contaminant degradation. This dual functionality not only reduces costs but also contributes to mitigating environmental pollution. Nevertheless, the challenge lies in the quest for photocatalysts capable of effectively facilitating both reactions concurrently, which constitutes a pivotal research area. Distinct pollutants exhibit unique behaviors, necessitating varying catalyst materials. Achieving bidirectional selectivity between the photocatalyst and pollutant is of paramount importance in achieving optimal performance.

## Conflicts of interest

There are no conflicts to declare.

## Supplementary Material
